# Effectiveness of Internet-Based Electronic Technology Interventions on Breastfeeding Outcomes: Systematic Review

**DOI:** 10.2196/17361

**Published:** 2020-05-29

**Authors:** Alaa Ali Almohanna, Khin Than Win, Shahla Meedya

**Affiliations:** 1 School of Computing and Information Technology University of Wollongong Wollongong Australia; 2 School of Nursing University of Wollongong Wollongong Australia

**Keywords:** breastfeeding, mobile app, mobile phone, mHealth, internet, computers, systematic review

## Abstract

**Background:**

Supporting women to initiate and continue breastfeeding is a global challenge. A range of breastfeeding interventions employing electronic technologies (e-technologies) are being developed, which offer different delivery modes and features over the internet; however, the impact of internet-based e-technologies on breastfeeding outcomes remains unclear.

**Objective:**

This study aimed to identify the characteristics of current internet-based breastfeeding interventions employing e-technologies and investigate the effects of internet-based e-technologies on breastfeeding outcomes.

**Methods:**

A systematic search was conducted in accordance with the Preferred Reporting Items for Systematic Reviews and Meta-Analyses guidelines in the following databases: Scopus, Web of Science, the Cochrane Database of Systematic Reviews, ScienceDirect, Google Scholar, the Association for Computing Machinery, SpringerLink, and Institute of Electrical and Electronics Engineers Xplore.

**Results:**

This systematic review included 16 studies published between 2007 and 2018, with 4018 women in 8 countries. The characteristics of the interventions were grouped based on (1) mode of delivery (web-based, mobile phone apps, and computer kiosk), (2) purpose of the interventions (education and support), and (3) key strategies (monitoring and breastfeeding tracking, personalization, online discussion forum, web-based consultation, and breastfeeding station locators). Combining educational activities with web-based personalized support through discussion forums appeared to be the most effective way to improve breastfeeding outcomes and long-term exclusive breastfeeding rates. Monitoring and breastfeeding trackers appeared to be the least effective ways.

**Conclusions:**

This study demonstrated a variety of internet-based e-technologies that professionals can use to promote, educate, and support breastfeeding women. Future internet-based breastfeeding interventions employing e-technologies might consider improving interaction with mothers and personalizing the content of the proposed interventions.

## Introduction

Breastfeeding provides survival, growth, and health benefits for infants and promotes positive maternal health outcomes [1]. The United Nations Children’s Fund and the World Health Organization (WHO) refer to breastfeeding as a cornerstone for child survival, nourishment, and growth and maternal health [2]. However, only 38% of infants aged under 6 months are exclusively breastfed worldwide, which is lower than the 2025 target of 50% [3]. In Australia, 96% of women initiate breastfeeding after birth, but the feeding practices significantly declined to only 15% to 25% at 6 months postnatally [4].

Early breastfeeding cessation has attracted the attention of clinicians, health care providers, and governments to develop and assess new initiatives. The main interventions to promote and support breastfeeding include home visit counseling [5], peer counseling [6], peer support groups [7], and in-hospital educational intervention [8]. Breastfeeding education with support that starts from early pregnancy and continues to the late postnatal period was demonstrably one of the most effective interventions in long-term breastfeeding behavior [9]. However, providing traditional face-to-face education and support may require trained professionals [10], and it may not be easy for women and their families to attend educational sessions at an inconvenient time or place [11]. Hence, it has been argued that such traditional forms of support and education may not be beneficial or useful for younger generations [12]. Therefore, information and communications technology (ICT) may be suitable in transforming traditional education and support into a free and widely accessible mode of delivery [12,13].

ICTs are defined as digital technology tools and resources used to capture, handle, store, and exchange information via electronic communication [14]. The WHO indicated that ICT improves access to information, which will lead to an improvement in health care services around the world [15]. A study on mobile phone apps used by low-income participants in a public health nutrition program for Women, Infants, and Children in the United States revealed the need for improving and expanding technology in their program [3]. An international board–certified lactation consultant, Heinig [12], proposed that using the internet to deliver breastfeeding interventions is a way forward to promote breastfeeding. From April 2019, almost 4.4 billion people were actively using the internet [16]. Electronic technologies (e-technologies) are regarded as a revolutionary advance for providing health care services. The varied applications of e-technologies demonstrate improved operational efficiency and optimized time and productivity for both patients and health care professionals [10]. E-technologies such as web-based technologies, mobile apps, and computer kiosks use a broader range of ICTs to extend beyond the traditional health care facilities and provide support to geographically distant populations [17-19]. Evidence demonstrates that e-technologies can also deliver personalized web-based interventions that generate a longer-lasting health behavior change [20,21], such as in breastfeeding. Studies have shown a noticeable interest in more web-based options with personalized information, providing support to women on breastfeeding decisions [22-24]. Providing internet-based customizable support to mothers seeking information and help during their breastfeeding experience through e-technologies has the potential to impact any breastfeeding outcomes positively [25,26].

Many systematic reviews have assessed the efficacy of interventions employing e-technologies on maternity care and pregnancy outcomes [27-31]. However, only a few systematic reviews have reported on the effect of interventions employing e-technologies on breastfeeding outcomes. For instance, in a systematic review with 3 electronic interventional studies, using electronic-based interventions had a moderate effect on breastfeeding compared with provider-based interventions (OR 2.2, 95% CI 1.9-2.7; d=0.5 versus OR 1.1, 95% CI 1.0-1.2; d=0.0) [32]. Similarly, Giglia and Binns [33] reviewed references published between 2000 and May 2013 to assess the effect of using the internet on breastfeeding outcomes. The study found that among 1379 studies, only 1 study was eligible for inclusion and demonstrated a positive effect of using the internet on breastfeeding outcomes. The findings of another study on the efficacy of e-technologies in improving breastfeeding outcomes [34] suggested that interventions employing e-technologies are potentially valuable for improving breastfeeding knowledge, initiation, and duration of exclusive breastfeeding. However, there is no current systematic review to identify the different types of contemporary internet-based interventions employing e-technologies and assess their impact on breastfeeding outcomes. This study aimed to identify the features of the current internet-based breastfeeding interventions employing e-technologies and investigate the effects of internet-based e-technologies on breastfeeding outcomes.

## Methods

### Search Methodology

The Preferred Reporting Items for Systematic Reviews and Meta-Analyses (PRISMA) guidelines were followed in this review [35]. A PRISMA checklist and search terms are available in Multimedia Appendix 1.

### Information Sources

A total of 8 electronic databases were searched to identify potential studies: Scopus, Web of Science, the Cochrane Database of Systematic Reviews, ScienceDirect, Google Scholar, the Association for Computing Machinery, SpringerLink, and Institute of Electrical and Electronics Engineers Xplore. The registers of the following trials were also searched to identify any existing relevant trials: Cochrane Central Register of Controlled Trials, WHO International Clinical Trials Registry Platform, International Clinical Trials Registry Platform, and ClinicalTrials.gov.

### Types of Studies and Inclusion/Exclusion Criteria

Peer-reviewed studies, including quantitative and quality research, mixed methods, descriptive studies, randomized controlled trials, and quasi-experimental design trials with or without blinding, were included in the review. The research methodology was not limited in any way. Any papers examining interventions employing e-technologies that required internet access and aimed at addressing any breastfeeding outcome were included. Considering that SMS messaging can also be used without internet access, studies that used SMS messaging have been excluded from the review. The inclusion and exclusion criteria are shown in Textboxes 1 and 2.

**Inclusion criteria.**

- Topic
  - Papers evaluating any internet-based breastfeeding interventions employing electronic technologies (e-technologies; web-based, computer kiosk, and mobile app)
  - Papers evaluating internet-based breastfeeding interventions employing e-technologies among women
  - Papers explaining, assessing, or reporting any internet-based breastfeeding interventions employing e-technologies with any breastfeeding outcome
- Settings
  - No restriction
- Type of publications
  - Papers published in peer-reviewed journals or peer-reviewed papers from an international academic conference or conference proceeding.
- Language
  - English
- Publication date
  - No restriction

**Exclusion criteria.**

- Topic
  - Papers evaluating other breastfeeding interventions employing electronic technologies (e-technologies; eg, phone call, text messaging, and video or phone call)
  - Papers evaluating any internet-based breastfeeding interventions employing e- technologies targeted only at other relevant parties (eg, fathers, clinicians, providers, and health care worker or services)
  - Papers do not report on any breastfeeding outcome but focus on general maternal and child health
- Settings
  - No restriction
- Type of publications
  - Oral presentations, commentaries, policy briefs, and papers that described an app without evaluating its implementation and study protocol.
- Language
  - Not English
- Publication date
  - No restriction

### Types of Participants

Participants were healthy pregnant or postnatal women, either primiparous or multiparous, who intended to breastfeed. In addition, studies targeting both parents as study participants were also included if the intervention targeting women was described separately. Women of all ages, ethnicity, occupation, and any socioeconomic status were included.

### Search Strategy

The search was conducted on November 2018, whereas the screening stages occurred between January and March 2019. The following main key terms were used in the search strategy: nursing, breast-feed, “breast-feeding,” “breast feeding,” “breast milk” and lactation; and computers, telehealth, computer-mediated and mobile application. A detailed example of the search strategy tailored for Scopus is outlined in Textbox 3. This search strategy was adapted for each of the other databases (Multimedia Appendix 2).

**Search strategy for the Scopus database.**

(TITLE-ABS-KEY(Technology OR computer* OR web OR internet OR mobile* OR smartphone OR SMS OR video OR messag* OR application OR intervention OR promotion OR support OR cellphone OR ios OR android OR Cell* OR telephone OR text )) AND (TITLE-ABS-KEY(“Mobile health” OR mhealth OR m-health OR e-health OR ehealth OR telemedicine OR Telehealth OR Telelactation OR Health Information Technology OR behavio*)) AND (TITLE-ABS-KEY(Breastfeed* OR Breast-feed OR “Breast feed” OR lacta* OR “nursing mother*” OR mother* OR maternal health OR maternal care OR pregnan* OR antenatal OR post* OR newborn* OR infant* OR child* OR baby OR Exclusive Breast Feeding OR Exclusive Breastfeeding))

### Data Extraction

**Figure 1:**
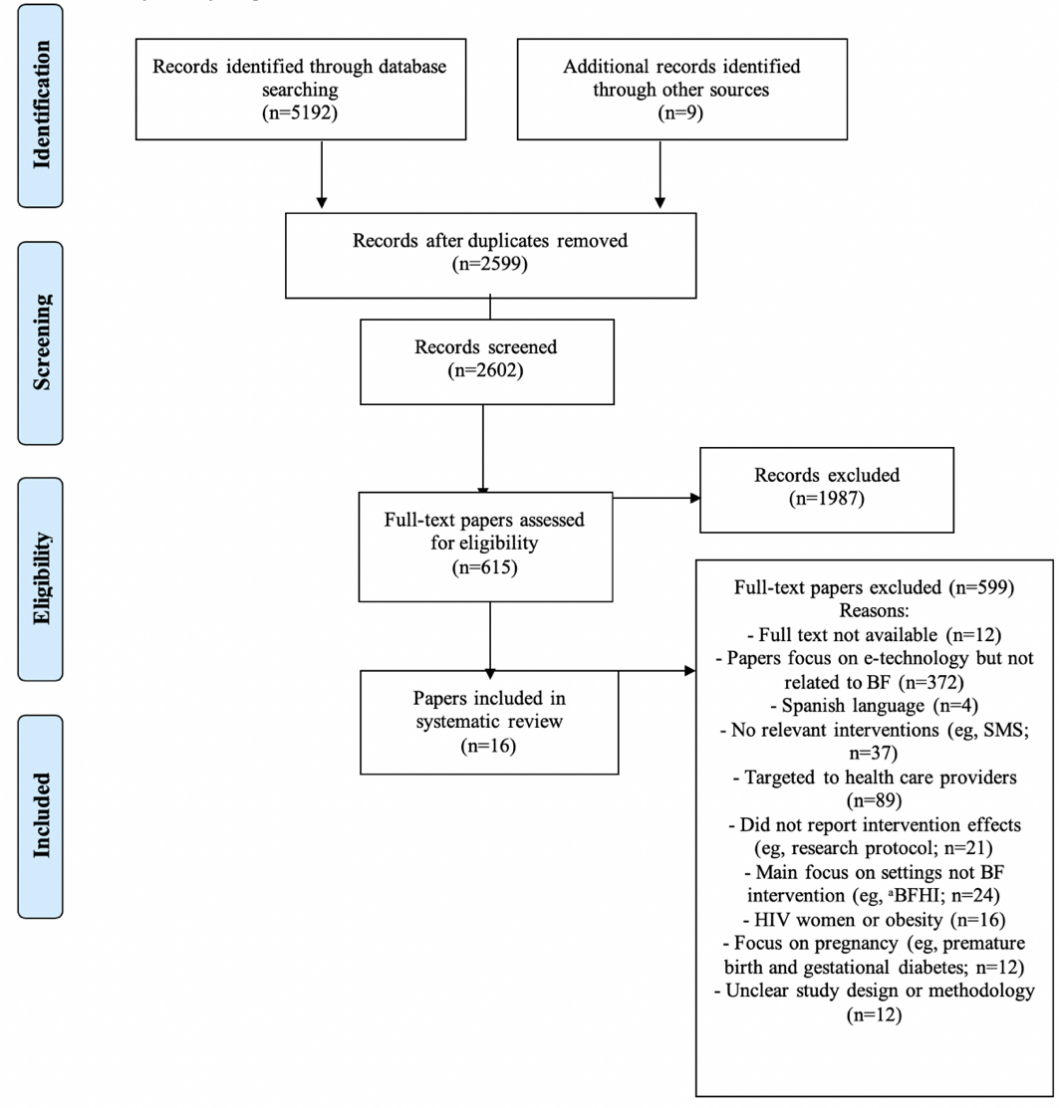
Flow Diagram of the systematic review - data extraction (BF, BSES). BF: breastfeeding; BHFI: baby friendly hospital initiative.

The retrieved studies for the search were transferred to Elsevier’s Mendeley Desktop Reference Manager by the first author (AA) to download papers and remove duplicates. The first author screened all titles and abstracts of the papers, and the full texts were reviewed to identify eligible studies. Then, 3 authors (AA, SM, and KW) discussed the suitability of the eligible studies for the final review. Studies that investigated maternal depression, HIV/Hepatitis C virus, smoking, diabetes, alcohol, overweight/obesity, fertility, prematurity, or cesarean section were excluded from the review. A table of the excluded studies with reported reasons is included in Multimedia Appendix 5. A diagram of the systematic review and data extraction is presented in Figure 1.

### Data Analysis

A self-adaptive narrative synthesis approach with thematic analysis [36] was used to categorize the purpose of internet-based interventions employing e-technologies and the strategies that were used in those interventions. Meta-analysis could not be processed because of the substantial heterogeneity existing among the studies. However, breastfeeding outcomes are presented in a narrative form.

### Search Outcome and Quality Appraisal

The search identified 5201 papers. After excluding duplicates, 2599 papers were screened for eligibility and 615 remained (Figure 1). After examination of the full texts, 17 studies fully met the inclusion criteria. The quality of the studies was appraised by 2 researchers (AA and SM), using the mixed methods appraisal tool [37,38]. After the quality appraisal, 1 study was excluded [39], and a total of 16 studies remained (Figure 1). Any disagreement on quality checks was resolved through discussion between the 3 authors (AA, SM, and KW). Tables showing the quality appraisal process are presented as additional files in Multimedia Appendix 4.

## Results

### Description of Studies

The included studies involved 4018 participants from 8 different countries: the United States (n=5), Australia (n=3), Finland (n=3), Taiwan (n=1), Canada (n=1), Thailand (n=1), the Philippines (n=1), and Ireland (n=1). The interventions in the included studies were delivered to women prenatally or postnatally, and all reported one or more breastfeeding outcomes (Table 1). Only 3 studies reported using a theoretical framework to inform the design of the intervention: the sustained breastfeeding framework based on predicting and changing behavior theory [40], Bandura’s [41] social cognitive theory [42], and Bandura’s [43] self-efficacy theory [44]. The description of the included studies is provided in Multimedia Appendix 3.

### Characteristics of Internet-Based Electronic Technology Interventions

Characteristics of the internet-based e-technology interventions in the 16 included studies were categorized by the mode of e-technology delivery, purpose of the intervention, and key strategies that were used in those interventions.

#### Mode of Electronic Technology Delivery

Electronic technology in the included studies involved a range of delivery approaches such as web-based interventions [42,44,46-54], mobile apps [55-57], and computer kiosk [40,45] (Table 2).

#### Purpose and Key Strategies Used in the Interventions

The purpose and key strategies used in the studies were coded using a thematic analysis. Overall, 2 main categories emerged based on the purpose of the interventions: breastfeeding education and breastfeeding support. On the basis of key strategies that were used in the interventions, 6 categories were identified: (1) monitoring and breastfeeding tracking, (2) personalization, (3) online discussion forum, (4) web-based consultation, and (5) breastfeeding station locator (Table 3).

### Breastfeeding Outcome Measures

Breastfeeding intention, knowledge, exclusivity, and duration were the most reported outcome measures in the studies (Table 4).

**Table 1:** Characteristics of included studies.

Study reference	Setting, study country	Study design	Participants	Duration	Breastfeeding outcome measure	Theoretical framework	Assessment tool
Joshi et al, 2016 [40]	The Regional West Medical Centre in Scottsbluff, United States	Quasi-experimental study	46 women	Antenatal to 6 months postnatal	Breastfeeding knowledge Breastfeeding self-efficacy Breastfeeding intention	Predicting and changing behavior theory	Breastfeeding knowledge questionnaire BSES-SF^a^ questionnaire Breastfeeding attrition prediction tool questionnaire
Zhang et al, 2014 [45]	Melrose-Wakefield Hospital in Massachusetts, United States	RCT^b^	15 women	Antenatal to 6 months postnatal	Breastfeeding knowledge Breastfeeding intention	NR^c^	Questionnaire BSES-SF
Ahmed et al, 2012 [46]	Two Midwestern hospitals, United States	Mixed methods	26 women	Postnatal to 30 days	Breastfeeding support Breastfeeding education	NR	Breastfeeding diary System usability scale Perception survey
Ahmed et al, 2016 [42]	3 hospitals in the Midwestern, United States	RCT	141 women	Postnatal to 3 months	Exclusive breastfeeding Breastfeeding duration Breastfeeding intensity	Social cognitive theory	Paper-based forums Surveys (online follow-up forums)
Alberdi et al, 2018 [47]	The National Maternity Hospital (Dublin, urban) and Wexford General Hospital (Wexford, rural), Ireland	Feasibility study	100 women	Antenatal to 3 months postnatal	Breastfeeding duration	NR	Web-based questionnaires
Geoghegan-Morphet et al, 2014 [48]	Hospital, Canada	Qualitative study	200 women	Postnatal to 6 months	Breastfeeding education Breastfeeding outcomes Breastfeeding support	NR	Surveys
Giglia et al, 2015 [49]	Hospitals from 4 regional areas, Australia	Longitudinal cohort study	414 women	Postnatal to 12 months	Breastfeeding support Breastfeeding initiation Breastfeeding duration Exclusive breastfeeding	NR	Modified tool based on Perth infant feeding study mark II
Grassley et al, 2017 [50]	Website, United States	Cohort study (1 group pre + post)	41 women	Antenatal to 1 month postnatal	Breastfeeding self-efficacy Breastfeeding intention Breastfeeding education	NR	Questionnaire (pretest and posttest) BSES-SF
Hannula et al, 2014 [51]	3 public maternity hospitals in the Helsinki Metropolitan area, Finland	Quasi-experimental study	705 women	Antenatal to first week postnatal	Exclusive breastfeeding Breastfeeding attitude Breastfeeding confidence Breastfeeding coping	NR	Iowa infant feeding attitude scale BSES-SF LATCH^d^ assessment tool
Huang et al, 2007 [52]	Hospital in Taipei, Taiwan	Quasi-experimental study	120 women	Antenatal to 6 weeks postnatal	Breastfeeding duration Breastfeeding knowledge Breastfeeding attitude	NR	Questionnaire
Newby et al, 2015 [53]	Feeding Queensland Babies, Australia	Prospective cohort study	488 women	Antenatal to 12 months postnatal	Breastfeeding support Breastfeeding education	NR	Web-based questionnaires
Salonen et al, 2008 [44]	Two public university hospitals, Finland	Quasi-experimental study	863 women	Antenatal to hospital discharge	Exclusive breastfeeding	Self-efficacy theory	Questionnaire
Salonen et al, 2014 [54]	Hospital, Finland	Quasi-experimental study	760 women	Antenatal to 12 months postnatal	Exclusive breastfeeding	NR	Structured questionnaire
Dela Cruz et al, 2017 [55]	The Philippine Human Milk Bank, Philippines	Qualitative study	32 women	NR	Breastfeeding support Breastfeeding education	NR	Web-based questionnaires
Wang et al, 2018 [56]	Website, Thailand	Mixed method	21 women	Postnatal to 4 weeks	Breastfeeding support Breastfeeding education	NR	Surveys Structured interview
Wheaton et al, 2018 [57]	4 local hospitals at Southwest Victoria, Australia	Prospective cohort study	46 women	Postnatal to 6 months	Breastfeeding duration Breastfeeding confidence	NR	Web-based questionnaires

^a^BSES-SF: breastfeeding self-efficacy scale—short form.

^b^RCT: randomized controlled trial.

^c^NR: not reported.

^d^LATCH: breastfeeding charting system and documentation tool.

**Table 2:** Description of internet-based electronic technology interventions.

Study reference	Type of technology intervention	Purpose/objectives	Providers
Joshi et al, 2016 [40]	Computer kiosk	To provide breastfeeding knowledge and enhance self-efficacy through an interactive computer kiosk and a bilingual breastfeeding educational program	Nurses
Zhang et al, 2014 [45]	Computer kiosk	To promote breastfeeding through a virtual lactation consultant on a computer kiosk	University staff
Ahmed et al, 2012 [46]	Website	To provide breastfeeding education and support through an interactive web-based breastfeeding monitoring system (LACTOR)	Trained research team Lactation consultants
Ahmed et al, 2016 [42]	Website	To increase breastfeeding duration, exclusivity, and intensity through a web-based interactive breastfeeding	Research scientist and a lactation consultant
Alberdi et al, 2018 [47]	Website	To assess breastfeeding initiation and duration using a web-based breastfeeding support	Lactation specialists
Geoghegan-Morphet et al, 2014 [48]	Website	To provide breastfeeding education with integrated peer and professional support through a web-based breastfeeding support clinic	Academic Medical Organization of Southwestern Ontario study team
Giglia et al, 2015 [49]	Website	To provide breastfeeding support using a website	Midwives and/or research staff Nurses
Grassley et al, 2017 [50]	Website	To provide breastfeeding education, assess breastfeeding intention, and promote breastfeeding self-efficacy using a web-based game-based learning platform	Researchers’ university
Hannula et al, 2014 [51]	Website	To provide an intensified support for breastfeeding using a web-based service	Midwives
Huang et al, 2007 [52]	Website	To provide breastfeeding education, increase breastfeeding knowledge, and enhance breastfeeding skills using a web-based breastfeeding education program	Midwives
Newby et al, 2015 [53]	Websites	To provide breastfeeding and education and support using web-based breastfeeding support and evaluate internet sources of infant feeding information	The researcher
Salonen et al, 2008 [44]	Website	To support breastfeeding, parenting, and infant care through a web-based information website	Nurses
Salonen et al, 2014 [54]	Website	To support breastfeeding, parenting, and infant care through a web-based information website	Nurses
Dela Cruz et al, 2017 [55]	Mobile app	To provide breastfeeding education and support using a mobile app	The researchers
Wang et al, 2018 [56]	Mobile app	To provide breastfeeding education and support through a mobile phone app	The researchers
Wheaton et al, 2018 [57]	Mobile app	To evaluate breastfeeding duration compared with the general population and describe infant feeding outcomes	Nurses and/or midwives

**Table 3:** Classification of the internet-based electronic technologies based on the purpose of the interventions and the key strategies.

Purpose and key strategies		Computer kiosk			Website												Mobile app		
		Joshi et al, 2016[40]	Zhang et al, 2014 [45]	Ahmed et al, 2016 [42]		Huang et al, 2007 [52]	Hannula et al, 2014 [51]	Salonen et al, 2008 [44]	Salonen et al, 2014 [54]	Geoghegan-Morphet et al, 2014 [48]	Grassley et al, 2017 [50]	Ahmed et al, 2012 [46]	Giglia et al, 2015 [49	Alberdi et al, 2018 [47]	Newby et al, 2015 [53]	Wang et al, 2018 [56]		Dela Cruz et al, 2017 [55]	Wheaton et al, 2018 [57]
**Purpose**																			
	Education	✓^a^	✓	✓		✓	✓	✓	✓	✓	✓	✓	✓	✓	✓	N/A^b^		✓	✓
	Support	✓	✓	✓		✓	✓	✓	✓	✓	✓	✓	✓	✓	✓	✓		✓	✓
**Key strategies**																			
	Monitoring and breastfeeding tracking	N/A	✓	✓		N/A	N/A	N/A	N/A	N/A	N/A	✓	N/A	N/A	N/A	✓		N/A	N/A
	Personalization	✓	✓	✓		N/A	N/A	N/A	N/A	✓	N/A	✓	✓	N/A	N/A	✓		✓	✓
	Online discussion forums	N/A	N/A	N/A		✓	N/A	✓	✓	✓	N/A	N/A	✓	✓	✓	N/A		N/A	N/A
	Web-based consultant	N/A	N/A	✓		N/A	N/A	N/A	N/A	✓	N/A	✓	✓	N/A	N/A	N/A		N/A	N/A
	Breastfeeding stations locator	N/A	N/A	N/A		N/A	N/A	N/A	N/A	N/A	N/A	N/A	N/A	N/A	N/A	✓		✓	N/A

^a^**✓**: applicable.

^b^N/A: not applicable.

**Table 4:** Reported breastfeeding outcomes based on the types of internet interventions employing electronic technologies.

Outcome measure^a^	Total (N=16), n (%)	Computer kiosk, n	Website, n	Mobile app, n
Breastfeeding initiation	1 (6)	N/A^b^	1	N/A
Exclusive breastfeeding	5 (31)	N/A	5	N/A
Breastfeeding duration	5 (31)	N/A	4	1
Breastfeeding intention	3 (19)	2	1	N/A
Breastfeeding knowledge	3 (19)	2	1	N/A
Breastfeeding attitude	1 (6)	N/A	1	N/A
Breastfeeding confidence	2 (13)	N/A	1	1
Breastfeeding self-efficacy	1 (6)	1	N/A	N/A
Breastfeeding intensity	1 (6)	N/A	1	N/A

^a^Studies with multiple outcomes were counted repeatedly in each electronic technology category.

^b^N/A: not assessed.

### Breastfeeding Outcomes With Web-Based Interventions

Of 11 web-based breastfeeding interventions that had a combination of education and support focus, 8 demonstrated improvements in breastfeeding outcomes [42,44,47-49,51,52,54].

For instance, Huang et al [52] evaluated breastfeeding knowledge in a quasi-experimental study (n=120) where there was a significant difference in breastfeeding knowledge level between the intervention and control groups in the posttest results (*P*<.001), with no differences in the pretest knowledge level. In addition, the exclusive breastfeeding rates were statistically higher at 3 to 5 days and at 2, 4, and 6 weeks in the intervention group (n=60) compared with the control group (n=60; 48.3%, 45%, 31.7%, and 26.7% versus 38.3%, 20%, 20%, and 20%, respectively) [52].

Exclusive breastfeeding during hospital stay has been shown to improve with web-based interventions [44,51,54]. Hannula et al [51] evaluated the effect of a web-based breastfeeding educational support system on exclusive breastfeeding rates in 705 women in Finland. The intervention group (n=431) had access to a website that offered articles, pictures, videos, and an educational game. During hospital stay, the intervention group breastfed exclusively more often than the control group (71% versus 58%; *P*<.001) and likewise on discharge (76% versus 66%; *P*=.01). In addition, 2 other quasi-experimental studies conducted in Finland with a convenience sample of 1300 [44,54] also indicated higher exclusive breastfeeding rates in a group of women who used web-based interventions compared with the control group during their stay in the hospital. Salonen et al [44] reported that exclusive breastfeeding was more common in the intervention group (51.0% versus 27.4%; *P*<.001) and similar findings were also reported by Salonen et al [54] (50.1% versus 30.4%; *P*<.001).

Providing web-based breastfeeding intervention with an interactive and asynchronous online discussion board was found useful in motivating intervention mothers to continue breastfeeding for longer periods of time [49]. For instance, in a longitudinal cohort study among regional Western Australian women, using online support and discussion forums was associated with higher exclusive breastfeeding rates at 6 months among women who lived in remote areas (n=10; 5.9% versus 0.6%; *P*=.01) [49].

Access to a website with information on breastfeeding has also been offered to 127 women in a recent study in Ireland along with sending emails up to 6 months postpartum and providing access to a breastfeeding helpline and support group [47]. The study demonstrated a positive impact on breastfeeding duration, as participants from both urban and rural areas acknowledged an increase in breastfeeding duration because of participation in the study, 42.2% and 86.7%, respectively. However, urban women (42% at 6 weeks and 53.3% at 3 months) reported no impact on the length of time they breastfed from participation in the study, compared with rural women (26.7% at 6 weeks and 13.3% at 3 months). This study found that providing additional support postnatally, including exclusive access to the educational website, was more preferred by women in rural areas compared with those in urban areas, with 46.7% and 22%, respectively [47].

Geoghegan-Morphet et al [48,58] designed a web-based breastfeeding support clinic called the Maternal Virtual Infant Nutrition Support clinic to offer evidence-based breastfeeding education combined with peer and professional support. The resource had 4 aspects: (1) text, graphics, and video breastfeeding educational resources; (2) an interactive discussion forum for participants, which is supervised and facilitated by an international board–certified lactation consultant; (3) data collection capacity; and (4) a web-based infant journal for text and photo entries. The study found that 60.8% of mothers were exclusively breastfeeding at 6 months postpartum in the intervention group compared with 28.2% of mothers, similar to statistics from the Canadian 2010-2013 data for Ontario mothers [58].

A US-based pilot study [50] designed the *HealthyMoms* intervention aimed to educate women about breastfeeding using a game-based learning platform (3D Gamelab) on the web. A total of 3 quests on the 3D Gamelab platform were completed by the study participants and focused on three main topics: deciding about breastfeeding, feeding your baby, and getting support. Each one of the quests focused on a specific breastfeeding topic using web-based education activities, such as watching a video, reading a short introduction, or adding posts and responses to the information. Breastfeeding self-efficacy was measured with the 14-item breastfeeding self-efficacy scale—short form (BSES-SF), and breastfeeding intention was assessed using a 1-item measure with 4 categories (eg, *just breastfeed/no formula*, *just formula/no breastfeeding*, *both*, or *unsure*). The majority of mothers (68%) reported a high intention for exclusive breastfeeding; however, a one-way analysis of variance test found no significant differences among the groups in mean BSES-SF scores before (*P*=.26) or after (*P*=.68) the intervention [50].

Furthermore, 2 studies evaluated the impact of web-based breastfeeding monitoring systems among breastfeeding mothers [42,46]. Of the systems, one [42] found no significant differences in breastfeeding outcomes between the women in the intervention group (n=49) and the control group (n=57) upon discharge (*P*=.71). However, the women in the intervention group had better exclusive breastfeeding rates (63%, 63%, and 55%, respectively) at 1, 2, and 3 months, respectively, compared with the control group (40%, 19%, and 19%, respectively). Participants (n=26) in a study by Ahmed and Ouzzani [46] also received postdischarge breastfeeding support through web-based breastfeeding monitoring systems and reported that the system helped them to minimize breastfeeding problems [46].

### The Key Strategies Used in the Web-Based Interventions

#### Monitoring and Breastfeeding Tracking in the Web-Based Interventions

Only 2 studies [42,46] provided web-based breastfeeding diary interventions and used data monitoring strategies to promote breastfeeding.

Providing a web-based breastfeeding diary [42] designed to record breastfeeding data for 30 days had a significant effect on breastfeeding intensity between the intervention and control groups at 3 months (*P*=.002). Ahmed and Ouzzani [46] evaluated the impact of a web-based breastfeeding monitoring system (LACTOR) designed to record breastfeeding and infant output data for 30 days. The system has 2 modules: (1) the mothers’ portal, where mothers can record their daily breastfeeding data and get notifications; and (2) the lactation consultant’s portal. The study found that more than 77% of the mothers reported infant feedings ≥8 times per day and reported that the system provided motivation to continue breastfeeding. Although 70% of mothers stated that breastfeeding data entry was not time-consuming, they reported that monitoring did not substitute face-to-face consultation with a lactation consultant.

#### Personalization in the Web-Based Interventions

Delivering personalized support was cited in four studies [42,46,48,49]. In all, two web-based breastfeeding monitoring systems [42,46], asked participating mothers to enter their breastfeeding data daily where both systems are capable of generating an automatic feedback sent via notifications with tailored interventions depending on the entered breastfeeding problem. These systems are capable of detecting patterns from responses of mothers and recognizing different breastfeeding problems. The system also provided motivation for mothers in the intervention group by sending a positive notification when a mother breastfed 8 to 10 times per day [42]. A website breastfeeding intervention in Australia enabled the intervention group (n=207) to post on discussion forums, start a new email conversation with others, and use a webcam to contact lactation consultants and receive a tailored response [49]. A more personalized intervention was proposed in a study by Geoghegan-Morphet et al [48], where a lactation consultant offered live help sessions through the web-based clinic and real-time response; however, these help sessions were not utilized by any of the participating women.

#### Online Discussion Forums in the Web-Based Interventions

Providing a web-based breastfeeding intervention with an interactive and asynchronous online discussion board was found to be effective in increasing rates of exclusive breastfeeding [52] and useful in motivating mothers in the intervention group to continue breastfeeding for longer [49]. Discussion forums were also used by mothers to contact a registered nurse/midwife to ask questions and receive credible information [44,54]. However, no relationship was discovered between accessing online chatrooms/discussion forums for breastfeeding support and providing breast milk for infants [53]. Remarkably, the breastfeeding helpline in an Irish cohort [47] was accessed by 30.6% of urban women and only 12.5% of rural women.

The web-based breastfeeding support clinic developed in a study in Canada [48] improved access to specialized professional breastfeeding support and offered communication and engagement through discussion forums. The intervention group received full access with peer and professional support in the discussion forum, and the control group received full access except for the interactive discussion forum [48].

#### Web-Based Lactation Consultant in the Web-Based Interventions

In an internet-based intervention study by Giglia et al [49], which was effective in long-term breastfeeding outcomes, women in the intervention group were provided with access to a certified lactation consultant through web-based posts or using a webcam with any of their concerns or questions regarding breastfeeding. Although women in this study reported several breastfeeding issues at each time point, none of them contacted a lactation consultant through webcam services.

Similarly, a web-based breastfeeding support clinic [48] offered support and educational resources to mothers facilitated by a lactation consultant where they could monitor, facilitate, and encourage discussions on the discussion forum. They could also post information and resources, set questions for additional details, answer questions, and offer suggestions. However, the post topics from the intervention group tended to be on more subjective (eg, lifestyle based) rather than technical topics (eg, breastfeeding problems) [48]. In 2 studies [42,46], the breastfeeding data of mothers were continuously monitored on the web by lactation consultants. Early detection and resolution of breastfeeding problems by lactation consultants presented improvements to breastfeeding continuation.

### Breastfeeding Outcomes With Using Mobile Apps

A total of 3 studies reported on the usability of mobile apps without examining the effectiveness of the app on breastfeeding outcomes [55-57]. One Australian study evaluated the usability of a smartphone breastfeeding app, *Breastfeeding Solutions*, among rural Australian breastfeeding women [57]. The app was an interactive guide to resolve breastfeeding problems, provide functions for searching for problem solutions, and deliver timely information for mothers. The results of the study demonstrated its usability by a longer duration of breastfeeding compared with general statistics from southwest Victoria [57].

A total of 2 other studies with small sample sizes evaluated the usability and usefulness of the mobile phone apps *MoomMae* [56] *and Milktrack* [55] designed to provide support to breastfeeding women. The *MoomMae* [56] intervention aims to support mothers in feeling more comfortable breastfeeding in public and efficiently track their feeding and pumping logs.

### Key Strategies Used in Mobile Device Apps

#### Monitoring and Breastfeeding Tracking in Mobile Apps

Only one breastfeeding mobile app intervention [56] offered breastfeeding tracking features to enter pumping/feeding data and save the history of all feeding records.

#### Personalization in Mobile Apps

Participants enrolled in 3 studies [55-57] received a personalized breastfeeding intervention through mobile apps. The interventions were designed to enhance the breastfeeding experience by providing feeding and pumping volume control [56], offering a platform to donate human milk [55], and providing an interactive guide to solving the breastfeeding problems of mothers [57].

#### Breastfeeding Station Locator in Mobile Apps

In all, 2 studies in Thailand [56] and the Philippines [55] developed mobile apps with breastfeeding station locators with an embedded strategy that can locate nearby places accessible for breastfeeding using the user’s GPS location. In both studies, women perceived that locating places for breastfeeding through the app was easier, but the *feeding room* feature still had some negative feedback because of the limited number of feeding rooms exhibited in the app [56]. 

### Breastfeeding Outcomes Using a Computer Kiosk

A total of 2 studies used computer kiosk interventions [40,45]. The main purpose of using computer kiosks was to provide breastfeeding education and support by incorporating several educational modules such as basics of breastfeeding, benefits of breastfeeding, and coping with breastfeeding [40,45]. A pilot evaluation study [45] showed significantly greater breastfeeding knowledge in the intervention group (n=7) after they interacted with the prenatal module (*P*<.05) in comparison with the control group (n=8). This study showed significantly greater intention to exclusively breastfeed after women interacted with the prenatal module (*P*<.05) and improvement in breastfeeding confidence compared with the control group [45]. Similarly, Joshi et al [40] reported a significant improvement in breastfeeding knowledge scores (*P*=.03) only at week 6 of the follow-up between the control (mean 23.2, SD 3.7) and intervention (mean 25.3, SD 2.6) groups. However, no significant differences were perceived in the average change in knowledge scores between the control and intervention groups at any other follow-up time points. This study also reported a gradual increase in the breastfeeding self-efficacy scores until week 6, followed by a decrease in self-efficacy scores at 3 months (*P=*.46) and 6 months (*P*=.54). Moreover, the intervention group reported significantly higher intention to breastfeed (*P*=.049), and the results indicated a significant improvement in breastfeeding intention scores over a 6-month period with all study participants (*P*<.05).

### Key Strategies Used in Computer Kiosks

#### Monitoring and Breastfeeding Tracking With Computer Kiosks

Only the computer kiosks in the Zhang et al [45] study had a longitudinal breastfeeding tracking feature, which enabled the system to examine breastfeeding records and monitor breastfeeding practices to ensure optimal infant growth.

#### Personalization With Computer Kiosk

Zhang et al [45] developed a virtual lactation consultant on a computer kiosk to interact with women in the intervention group. The intervention included motivational interviewing techniques to motivate and social cognitive techniques to reinforce positive behaviors. The intervention enabled adaptive interaction, where the interactive component was modifiable based on a mother’s own progress and her previous interactions with the kiosk. Joshi et al [40] designed an interactive kiosk to present breastfeeding information and messages adjustable depending on the psychosocial elements, including self-efficacy, influence of attitude, expectancies, personal norms, and social effect.

## Discussion

### Principal Findings

This study presents a review of internet-based breastfeeding interventions employing e-technologies; investigates their purpose, mode of delivery, and key strategies; and systematically describes their effectiveness on breastfeeding outcomes. The main purpose of the reviewed studies focused on education and support in 3 modes of delivery: web-based interventions, mobile apps, and computer kiosks. Among the 5 key strategies, personalization and web-based discussion were the most common strategies used in the interventions. This review provides evidence that internet-based interventions employing e-technologies that provide a combination of early interactive antenatal breastfeeding education with postnatal web-based discussion support can improve breastfeeding outcomes in hospital stay [44,47,48,51,52,54] and exclusive breastfeeding rates up to 6 months [49]. These results are supported by similar face-to-face interventional studies where there is a combination of antenatal education and postnatal support [59-61].

Interventions with personalized feedback and tailored information to mothers through their support systems were found to be motivational and led to positive breastfeeding outcomes. These effective strategies point toward a need for informed, highly interactive, and tailored-designed breastfeeding interventions employing e-technologies. Consistent with other effective clinical interventions, conducting multimodal, multiphased, and interactive interventions are successful in breastfeeding practices [60,62,63]. Web-based support could be offered informally, such as peer communication through online discussion forums, or formally, such as web-based lactation consultation. The interactivity, connectivity, and two-way communication provided between mothers and lactation consultants in breastfeeding interventions may expand the opportunities for educating and engaging mothers and thus improve breastfeeding outcomes. Providing access to timely and qualified lactation consultants in similar interventions has been found to be particularly attractive to mothers experiencing some breastfeeding complications [64]. In a Cochrane review of support interventions, the findings demonstrated that women who receive any form of support are less likely to stop exclusive breastfeeding before 5 months postpartum [65].

In this review, interventions that provided monitoring and breastfeeding tracking appeared to be least effective in improving breastfeeding outcomes, which could be because of a lack of meaningful educational and supportive interactions for different breastfeeding challenges. It is important to note that women in the included studies had higher prenatal breastfeeding intention rates, which could be a confounding factor in the effectiveness of the interventions [40,45,50]. Breastfeeding women are very likely to seek out extensive evidence-based information on breastfeeding information from reliable sources. The acquired breastfeeding knowledge is presumed to have positive influences on their feeding decisions [66].

In terms of the mode of delivery, web-based platforms were the dominant and more effective mode of delivering breastfeeding internet-based interventions employing e-technologies. Several studies used web-based interventions, and the majority of them reported an increase in exclusive breastfeeding after the intervention and overall positive outcomes in their results [42,44,47,49,51,52,54]. This finding may be explained by the ability of web-based interventions to sustain their effects in terms of providing early education and continued support to participants during the intervention from a variety of care providers (eg, peers and lactation consultants) in a range of settings (eg, hospital and home). Another reason could be related to the reliability and credibility of the governmental web-based platforms [67]. Women need to make informed decisions regarding the health and well-being of their babies. Having a trusted resource would empower women to make their own choices in breastfeeding. However, many studies have reported the lack of a regulatory system to assist end users in identifying the best available web-based e-technologies [68-70].

Although the delivery of breastfeeding interventions through mobile apps is encouraging because of their relative simplicity and continuous availability [71], the mobile apps in this review focused on supporting women to access milk banks or express their breastmilk among small groups of women. Furthermore, the studies mainly aimed at assessing the usability of the apps rather than examining their effectiveness without any theoretical framework. Although a significant absence in reporting a theoretical framework was observed in the majority of the included studies, none of the interventions with mobile apps reported using any type of behavior change theoretical framework. Using theory to inform intervention development or evaluation can play a key role in breastfeeding interventions as a strong predictor of breastfeeding behavior [72].

Finally, computer kiosk interventions seemed to be effective in enhancing women’s knowledge and short-term confidence level. Creating an interactive platform coupled with continuing, accessible support can aid mothers in having specific information to suit their personal needs [45,73]. The use of conversational computers could provide affordability and portability, as observed in other health-related interventions [74,75]. However, for long-term effects, there is a need to incorporate personalized approaches and professional support into interventions to create a practical response to the personal needs of mothers. In addition, sustainability remains an issue because these types of interventions could be outdated [76], and findings cannot be generalized to other settings.

A lack of detailed reporting of rigorous intervention development, evaluation, and the content of the implementation intervention was also observed, which makes it difficult to determine the impact of interventions on breastfeeding outcomes [77]. Future studies could design intervention implementation and evaluation guided by a theory toward a comprehensive intervention development process and for a better chance of effective breastfeeding intervention.

### Limitations

This systematic review has as a key strength that it followed the PRISMA guidelines [35], an established methodology for ensuring transparent reporting of systematic reviews. However, there are several limitations to this study; for example, it was difficult to generalize the findings observed from several studies because of their small sample size, different timing of studies, and lack of clarity on start and end points. The substantial heterogeneity of the intervention’s outcome measures and lack of clear definitions of breastfeeding outcomes in all the included studies added more complexity and prevented meta-analysis.

Another limitation of the study is that most of the included interventions were delivered and published in developed and high-income countries with good quality health care systems [78], which may not be generalizable to women in developing countries or those with diverse socioeconomic status and cultural background [29]. Setting up breastfeeding intervention in regions with inadequate health care providers and resources should be prioritized to improve breastfeeding practices [79]. Furthermore, the approaches of the interventions were focused on changing the behavior of individuals and lacked some key aspects related to cultural differences that may influence the breastfeeding practices of women and could impede them from achieving the desired outcome. This is particularly essential as the breastfeeding decisions women make differ according to whether their own culture is supportive of breastfeeding or not and on the cultural context in which they live. Thus, findings need further investigation regarding their transferability. Applicability needs to be considered when delivering breastfeeding interventions to women in different social milieu.

### Conclusions

The findings of the study demonstrate that internet-based e-technologies are transforming the access and delivery of breastfeeding interventions and that they have a considerable potential to assist breastfeeding mothers when seeking support and advice about breastfeeding. The results show that web-based interventions that provide a combination of education and ongoing support are the best models of interventions employing e-technologies to support long-term breastfeeding outcomes. In addition, the review reveals that the two dominant effective strategies are personalization, and online discussion forums form credible sources. Further studies need to explore the usability and effectiveness of interventions employing e-technologies that have theory-based systems designs that could incorporate encouragement and discussion opportunities from credible social and professional sources. The sociocultural needs of women also need to be integrated into these technologies to provide culturally tailored breastfeeding support.

## Appendix

Multimedia Appendix 1 Preferred Reporting Items for Systematic Reviews and Meta-Analyses 2009 checklist.

Multimedia Appendix 2 Database search strategies and keywords.

Multimedia Appendix 3 Table of the characteristics of included experimental studies.

Multimedia Appendix 4 Mixed methods appraisal tool, version 2018.

Multimedia Appendix 5 Excluded studies.

## Abbreviations

BSES-SF: breastfeeding self-efficacy scale—short form
e-technologies: electronic technologies
ICT: information and communications technology
PRISMA: Preferred Reporting Items for Systematic Reviews and Meta-Analyses
WHO: World Health Organization
