# Supplementary material for: Effectiveness of Internet-Based Electronic Technology Interventions on Breastfeeding Outcomes: Systematic Review
Source: J Med Internet Res. 2020 May 29;22(5):e17361. doi: 10.2196/17361 (PMC7293063; doi:10.2196/17361)
Supplement: Multimedia Appendix 2 [file jmir_v22i5e17361_app2.docx]

| Database | Results | Protocol, keywords or search strategy |
| --- | --- | --- |
| Google Scholar | 1500 | Find articles  with all of the words  breastfeeding AND technology ehealth |
| Scopus | 422 | (TITLE-ABS-KEY(Breastfeed* OR Breast-feed OR "Breast feed" OR lacta* OR "nursing mother*" OR mother* OR maternal* OR pregnan* OR antenatal OR post* OR newborn* OR infant* OR child* OR baby OR Exclusive Breast Feeding OR Exclusive Breastfeeding )) AND (TITLE-ABS-KEY(Technology OR computer* OR web OR internet OR mobile* OR smartphone OR sms OR video OR message OR application OR intervention OR promotion OR support OR cellphone OR ios OR android OR Cellular phone OR telephone OR text messag* OR short messag* OR cell phones OR short message service OR text )) AND (TITLE-ABS-KEY("Mobile health" OR mhealth OR m-health OR e-health OR ehealth OR telemedicine OR Telehealth OR Telelactation OR Health Information Technology OR behave*)) |
| ACM Digital Library | 11 | (+Breastfeeding) |
| SpringerLink | 712 | 'Breastfeed* OR Breast-feed OR “Breast feed” OR lacta* OR “nursing mother*” OR mother* OR maternal health OR maternal care OR pregnan* OR antenatal OR post* OR newborn* OR infant* OR child* OR baby OR Exclusive Breast Feeding OR Exclusive Breastfeeding AND Technology OR computer* OR web OR internet OR mobile* OR smartphone OR SMS OR video OR message OR application OR intervention OR promotion OR support OR cellphone OR ios OR android OR Cellular phone OR telephone OR text messag* OR short messag* OR cell phones OR short message service OR text AND “Mobile health” OR mhealth OR m-health OR e-health OR ehealth OR telemedicine OR Telehealth OR Telelactation OR Health Information Technology OR behavior' |
| Web of science | 805 | TS=(mhealth OR telemedicine OR telehealth OR ehealth OR m-health OR e-health OR Telehealth OR Telelactation OR "Health Information Technology"OR “Mobile health” OR persuasi* )  TS=(Technology OR computer* OR web OR internet OR mobile* OR smartphone OR SMS OR video OR message OR application OR intervention OR promotion OR support OR cellphone OR ios OR android OR Cellular phone OR telephone OR text messag* OR short messag* OR cell phones OR short message service OR text OR tablet OR digital device )  TS=(Breastfeed* OR Breast-feed OR “Breast feed” OR lacta* OR “nursing mother*” OR mother* OR perinatal OR postnatal OR newborn* OR infant* OR baby OR Exclusive Breast Feeding OR Exclusive Breastfeeding OR initiation OR duration )  #3 AND #2 AND #1  TS= (HIV OR depression OR smoking OR diabetes OR alcohol OR obesity OR premature OR vaccination OR weight lost OR physical activity OR Chemotherapy OR disease OR autism)  #4 NOT #5 |
| IEEE Xplore | 14 | (((breastfeed) OR "Author Keywords":breastfeed) OR "Abstract":breastfeed) |
| ScienceDirect | 1,219 | (breastfeeding) AND (app OR text OR computer OR technology OR mhealth OR Telelactation) NOT (hiv OR smok OR diabetes OR alcohol OR obesity OR premature OR vaccination OR weight lost OR physical activity OR Chemotherapy OR disease OR autism ) |
| The Cochrane Database of Systematic Reviews | 20 | 20 Cochrane Reviews matching on "breastfeeding" in Record Title - (Word variations have been searched) |
| Cochrane Central Register of Controlled Trials | 112 | 112 Cochrane Reviews matching on breastfeeding in Title Abstract Keyword  Years: 2010 – 2019 (Custom year range)  873 Trials matching on breastfeeding in Record Title |
| WHO International Clinical Trials Registry platform | 38 | Phases are: All Phase  With results only  38 records for 37 trials found for: breastfeeding |
| International Clinical Trials Registry Platform (ICTRP) | 21 | 21 records for 20 trials found for: breastfeeding |
| ClinicalTrials.gov | 318 | 318 Studies found for: Completed Studies \| breastfeeding |

**Multimedia Appendix: Search terms and studies retrieved**

### Search Terms

| Electronic technology  E-based technology / e-technology  Internet-based / internet / internet-based technology  Breastfeeding promotion  Duration initiation intention self-efficacy  Intervention  Technology  Computer mediated  Mobile application  Mobile health /mHealth/*e-Health*  Smartphone applications  Telemedicine / Telelactation |
| --- |
