# Supplementary material for: Effectiveness of Internet-Based Electronic Technology Interventions on Breastfeeding Outcomes: Systematic Review
Source: J Med Internet Res. 2020 May 29;22(5):e17361. doi: 10.2196/17361 (PMC7293063; doi:10.2196/17361)
Supplement: Multimedia Appendix 3 [file jmir_v22i5e17361_app3.docx]

## Multimedia Appendix 3

### Table of the Characteristics of included experimental studies

|  | Author Year | Purpose/Objectives | ﻿ Country/setting | ﻿Participants | Study Design | Intervention | Main Findings | BF Outcomes | ﻿Conclusion |
| --- | --- | --- | --- | --- | --- | --- | --- | --- | --- |
|  | Joshi, A. et al. (2016) | ﻿To evaluate the impact of an interactive, computer based, bi-lingual breastfeeding educational program on breastfeeding knowledge, self–efficacy and intent to breastfeed among rural Hispanic women living in Scottsbluff, Nebraska. | ﻿at the Regional West Medical Centre in Scottsbluff, Nebraska.  USA | ﻿46 rural Hispanic women | ﻿ Quasi -experimental study | ﻿﻿bilingual Computer based Breastfeeding Educational Support program | ﻿Improvement in breastfeeding knowledge, self-efficacy and intent to breastfeed was seen among individuals in the intervention group. | BF knowledge, self–efficacy and intent | Hispanic women living in rural settings showed improvement in breastfeeding knowledge, self-efficacy and intent to breastfeed using the computer based bi-lingual educational program |
|  | Zhang, Z. et al. (2014) | ﻿develop a virtual agent that promotes  breastfeeding from the third trimester to six months after birth | at Melrose-Wakefield Hospital in Massachusetts | ﻿15 women | Randomised controlled trial | Virtual agent  Computer | intervention group demonstrated significantly greater intent to exclusively breastfeed, and significantly greater breastfeeding knowledge following interaction with the perinatal module compared to the control group.  BF confidence | BF confidence  (BSES-SF 14-item 5-point scale)  BF knowledge (self-developed 1-item 7-point scale) | ﻿the breastfeeding promotion agent can have a real impact on postnatal breastfeeding practice. |
|  | Ahmed, A. H. et al. (2016) | ﻿whether a Web-based interactive breastfeeding monitoring system increased breastfeeding duration, exclusivity, and intensity as primary outcomes | ﻿Three hospitals in the Midwestern United States | ﻿(141) mother–newborn dyads were recruited before discharge | ﻿Two-arm, Randomised controlled trial | ﻿ online interactive breastfeeding monitoring system and were prompted to record breastfeeding and infant output data for 30 days. | -No significant differences in breastfeeding outcomes were found between groups at discharge  -Intervention group had greater exclusive breastfeeding rates at 1, 2, and 3 months  -By the end of the third month,84% of the intervention group was breastfeeding compared with 66% of the control group. | BF exclusivity, BF intensity | ﻿The Web-based interactive breastfeeding monitoring system may be a promising intervention to improve breastfeeding duration, exclusivity, and intensity. |
|  | Huang, M. Z. et al. (2007) | ﻿to evaluate a web-based breastfeeding education programme with the aim of deepening breastfeeding knowledge and enhancing skills. | ﻿from August 2003 to December 2003 at a hospital in Taipei, Taiwan | ﻿ ﻿control group (n = 60) or the experimental group (n = 60) | ﻿ Quasi -experimental design | ﻿web-based breastfeeding education | ﻿- breastfeeding knowledge and attitude are enhanced by web-based education programme  -﻿web-based education can increase the breastfeeding rate. | Exclusive BF initiation  Exclusive BF duration at 6 weeks  BF attitudes (BA: 26-item 5-point scale)  BF knowledge (BKT: 27-item 3-point scale) | web-based breastfeeding education may contribute to breastfeeding knowledge and attitude and improved breastfeeding rate. |
|  | Hannula, L. S., Kaunonen, M. E. and Puukka, P. J. (2014) | ﻿to assess the impact of providing intensified support for breast feeding during the perinatal period | three public maternity hospitals (two study, one control) in the Helsinki Metropolitan area in Finland. | ﻿705 mothers | Quasi-experimental | ﻿ web-based service intervention (﻿intensified breast-feeding support) | ﻿Intensified BF counselling and support helps mothers to breast feed exclusively.  - BF attitude  - Coping with BF  - BF confidence  BF initiation | Exclusive BF initiation  Breastfeeding attitudes (IIFAS 17-item 5-point scale)  Coping with BF (self-developed 8-item 5-point scale)  BF confidence (BSES-SF 14-item 5-point scale) | ﻿the low exclusive breast feeding rates of newborns could be increased by using intensified breast feeding support. |
|  | Salonen, A. H. et al. (2008) | ﻿to describe the development of an internet-based intervention for parents with infants and to compare the participants and hospitals at baseline. | Finland/﻿Two public university hospitals | ﻿total of 863 mothers  ﻿intervention (n = 469 mothers,  ﻿control (n = 394 mothers | Quasi-experimental | Web-based | ﻿-Exclusive breastfeeding was more common in the intervention hospital.  ﻿﻿-mothers in the intervention hospital were exclusively breastfeeding more often compared to mothers in the control hospital | Exclusive BF | ﻿The difference in the number of exclusively breastfed infants was also clinically significant |
|  | Salonen, A. H. et al. (2014) | ﻿evaluates how an internet-based intervention affects mothers' parenting satisfaction and infant centrality. | Finland/hospital | ﻿760 mothers. | Quasi-experimental | Web-based  ﻿online support for parenting and breast feeding | ﻿Intervention mothers were breast feeding exclusively more often than control mothers | Exclusive BF | ﻿an internet-based intervention did not significantly affect mothers' perceptions of parenting satisfaction and depressive symptoms, but intervention mothers experienced higher infant centrality |
|  | Geoghegan-Morphet, N. et al. (2014) | ﻿The development of this resource allows us to offer evidence-based breastfeeding education with integrated peer and professional support. It also lets us collect data on breastfeeding outcomes in a confidential on- line environment. | ﻿web site is held behind the London Health Sciences Centre hospital firewall. In Canada | ﻿200 participants  ﻿We recruited 200 healthy primiparous mothers of healthy term singletons during their postpartum hospital stay | ﻿links to surveys that appear at the data collection points | ﻿online breastfeeding support clinic  ﻿Maternal Virtual Infant Nutrition Support (MAVINS) Clinic | ﻿online breastfeeding support clinic has the potential to improve access to specialized professional breastfeeding support in combi- nation with interactive peer support. | BF support  BF education | The online delivery meets new mothers with a technology they are already using to seek health and parenting information. |
|  | Grassley, J.S., Connor, K.C. & Bond, L. 2017 | ﻿ to evaluate the effect of the Healthy Moms intervention on antenatal breastfeeding self-efficacy and intention and to determine the feasibility of using an online game-based learning platform to deliver antenatal breastfeeding education. | recruited using flyers from eight sites that provide care to women during pregnancy and from the study website.  USA | ﻿41 women | ﻿A pre-test post-test single group design was chosen to evaluate the effect of an online game-based learning intervention on breastfeeding self-efficacy and breastfeeding intention.  descriptive statistics and a one-way ANOVA. | ﻿The Healthy Moms intervention was designed using 3D Gamelab®, an online game-based learning platform.  Intervention on breastfeeding self-efficacy and breastfeeding intention | The pilot results suggest that Healthy Moms is a feasible method for  delivering breastfeeding information online  No significant differences in breastfeeding self- efficacy and intention were found among the groups | BF support  BF education | ﻿﻿Online antenatal breastfeeding education is feasible  ﻿computer-based education could be an efficient way to  deliver breastfeeding information  however, further research is warranted to determine if it can affect breastfeeding outcomes |
|  | Ahmed, A. H. and Ouzzani, M. (2012) | ﻿ Develop an interactive Web-based breastfeeding monitoring system (LACTOR) and examine its feasibility, usability, and acceptability among breastfeeding mothers. | ﻿mother infant units in 2 Midwestern hospitals in the United States. | ﻿Twenty-six mother/infant dyads | ﻿mixed-methods study | ﻿Interactive Web-based breastfeeding monitoring system (LACTOR) to Record Breastfeeding data daily for 30 days.  Online exit survey | ﻿ ﻿LACTOR is user-friendly, ﻿interactive and acceptable among mothers  ﻿usefulness of LACTOR system in recognizing infant feeding patterns | BF support  feasibility  Accessibility | ﻿LACTOR proved to be feasible, usable, and acceptable with features that help mothers navigate and recognize their breastfeeding problems |
|  | Giglia, R. et al. (2015) | ﻿to evaluate the effect of a breastfeeding support Internet intervention on breastfeeding outcomes on women living in regional Western Australia.  ﻿ | maternity ward level (maternity service in ﻿hospitals from four regional areas of Western Australia) by midwives and/or research staff or through regional Child Health Nurses during a universal home visit to new mothers within the first week post discharge | ﻿414 women | ﻿nested intervention design within a longitudinal cohort  titled the Regional Infant Feeding Study | ﻿Internet support Web site | ﻿Women enrolled in the intervention were significantly more likely to be exclusively breastfeeding at 6 months postpartum compared with control group participants. | BF initiation  BF duration | ﻿This breastfeeding support intervention study demonstrated a positive effect on longer-term exclusive breastfeeding for those enrolled in the intervention group. |
|  | Wang, C.-J., Chaovalit, P. and Pongnumkul, S. (2018) | ﻿to evaluate the usability and usefulness of MoomMae, a mobile phone app designed to support breastfeeding women. | ﻿Participants were approached via recruitment posters through two channels  ﻿Thailand | ﻿21 breastfeeding women | ﻿A pre-use interviews  Use the app for 4 weeks  A ﻿post-use interview | MoomMae, a breastfeeding mobile phone app | ﻿MoomMae has a great potential to be a useful self-management tool for breastfeeding mothers in Thailand  The qualitative analysis suggested that the app is supportive of breastfeeding on demand. | BF support  Usability  Usefulness | ﻿The quantitative results showed a high usability and usefulness score. The qualitative findings provided the insights into usability issues and usefulness of the app.  promotes self-efficiency |
|  | Dela Cruz, D. R. and Mendoza, D. M. M. (2017) | ﻿to reduce infant mortality rate as well as to prevent under-nutrition and stunted growth among children through empowering the breastfeeding practice | ﻿The Philippine Human Milk Banking Guidelines  Philippines | ﻿(32) mothers | ﻿  collect user experience information for  evaluating the mobile application.  Data collection was through ﻿  survey and questionnaires. | ﻿ Milktrack  Mobile Application | Convenience in  finding a place to  breastfeed or express breast milk is also achieved through the  capability of the application  to locate  nearby  breastfeeding stations. Providing relevant information about breastfeeding contributes in raising awareness thus encouraging more parents to practice breastfeeding | BF support  BF education | ﻿the development of the  Milktrack mobile application serves as an effective way of promoting and enhancing breastfeeding practice in the Philippines  ﻿  promoting breastmilk practice and awareness |
|  | Wheaton, N., Lenehan, J. & Amir, L.H. 2018 | ﻿Explore usability of the application among rural Australian breastfeeding women  ﻿Describe participants’ infant feeding outcomes compared with the general population  ﻿ | ﻿Southwest Victoria, Australia, ﻿across four rural local government areas, involving four local hospitals, from 2016 to 2017 | ﻿46 ﻿rural women | ﻿prospective longitudinal self-report survey design  ﻿online questionnaires  ﻿Descriptive statistics | breastfeeding  ﻿smartphone application | ﻿The app ﻿provided rural women with access to reliable and evidence- based information regarding breastfeeding.  the proportion of women continuing to  breastfeed was higher than the recent rates in the area. | BF Duration  ﻿BF Confidence  ﻿BF desirability | ﻿The smartphone application was acceptable to breastfeeding women in an Australian rural setting.  ﻿apps as a means of accurate, reliable, and timely information for mothers. |
|  | Alberdi, G. et al. (2018) | ﻿To evaluate the feasibility and acceptability of a multidimensional breastfeeding intervention in a rural and an urban maternity setting in Ireland.  ﻿ | ﻿The National Maternity Hospital (Dublin, urban) and Wexford General Hospital (Wexford, rural)  Ireland | ﻿100 women | ﻿ ﻿one-arm multicentre  feasibility study of a breastfeeding-support intervention. | ﻿-direct breastfeeding helpline with the lactation consultant for advice and/or help  - ﻿encouraging emails from the research team  - ﻿exclusive access to a study website with information on breastfeeding. | ﻿Data suggest that this type of intervention can improve current breastfeeding rates  BF duration | BF Initiation and BF duration | ﻿This multidimensional intervention is well-accepted and feasible to carry out within an Irish cohort, in both urban and rural areas. |
|  | Newby, R. et al. (2015) | ﻿to evaluate sources of infant feeding information used by first-time mothers and to describe breast and formula feeding patterns 6 months post birth associated with successful use of the Internet for breastfeeding support. | ﻿were recruited into the Feeding Queensland Babies Study by convenience sampling initially at a public event for families in Brisbane and by word of mouth and social and traditional media  ﻿Australia | ﻿488 women | ﻿ online questionnaire | ﻿online breastfeeding support | ﻿Women who sought infant feeding information on the Internet and reported success were 3 times more likely to be breast- feeding their infants and only one quarter as likely to be giving them formula at 6 months of age as mothers who sought but could not find the help they required online. | BF support | ﻿Online breastfeeding information and support may help women to meet their breastfeeding intentions and to minimize formula use. |
