# Supplementary material for: Effectiveness of Internet-Based Electronic Technology Interventions on Breastfeeding Outcomes: Systematic Review
Source: J Med Internet Res. 2020 May 29;22(5):e17361. doi: 10.2196/17361 (PMC7293063; doi:10.2196/17361)
Supplement: Multimedia Appendix 4 [file jmir_v22i5e17361_app4.docx]

## Multimedia Appendix 4

### Appraisal Tool

**Mixed Methods Appraisal Tool (MMAT), version 2018**

| [1] A. Joshi, C. Amadi, J. Meza, T. Aguire, and S. Wilhelm, “Evaluation of a computer-based bilingual breastfeeding educational program on breastfeeding knowledge, self-efficacy and intent to breastfeed among rural Hispanic women,” *Int. J. Med. Inform.*, vol. 91, pp. 10–19, Jul. 2016. | | | | | |
| --- | --- | --- | --- | --- | --- |
| **Category of study designs** | **Methodological quality criteria** |  | | **Responses** | |
|  |  | Yes | No | Can’t tell | Comments |
| Screening questions (for all types) | S1. Are there clear research questions? | Y |  |  |  |
|  | S2. Do the collected data allow to address the research questions? | Y |  |  |  |
|  | *Further appraisal may not be feasible or appropriate when the answer is ‘No’ or ‘Can’t tell’ to one or both screening questions.* | | |  | |
| 3. Quantitative nonrandomized | 3.1. Are the participants representative of the target population? | Y |  |  | ﻿46 women were enrolled and through simple randomization were assigned to Intervention and control groups. |
|  | 3.2. Are measurements appropriate regarding both the outcome and intervention (or exposure)? | Y |  |  | ﻿ ﻿Knowledge of breastfeeding was assessed using the breast-  feeding knowledge questionnaire (BKQ) and ﻿confidence in breastfeeding was gathered using the Breastfeeding Self-efficacy Scale Short Form (BSES-SF) questionnaire. ﻿ |
|  | 3.3. Are there complete outcome data? | Y |  |  | Missing responses were accorded a point of 0.5 multiplied by the weight for the particular subscale |
|  | 3.4. Are the confounders accounted for in the design and analysis? **This part is about the confounders that can influence the main outcome. For example age and education can influence breastfeeding rates. Did the author considered those elements. Happy to talk about this. Please send me your mobile number so I can call and we can discuss.** | 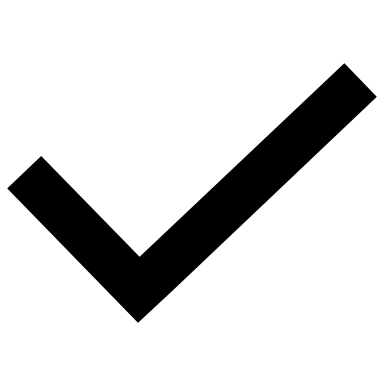 |  |  | no confounding expected (enrolment ﻿through simple randomization) |
|  | 3.5. During the study period, is the intervention administered (or exposure occurred) as intended? | Y |  |  |  |

| [1] Z. Zhang *et al.*, “Maintaining continuity in longitudinal, multi-method health interventions using virtual agents: The case of breastfeeding promotion,” *Lect. Notes Comput. Sci. (including Subser. Lect. Notes Artif. Intell. Lect. Notes Bioinformatics)*, vol. 8637 LNAI, pp. 504–513, 2014. | | | | | |
| --- | --- | --- | --- | --- | --- |
| **Category of study designs** | **Methodological quality criteria** |  | | **Responses** | |
|  |  | Yes | No | Can’t tell | Comments |
| Screening questions (for all types) | S1. Are there clear research questions? | Y |  |  |  |
|  | S2. Do the collected data allow to address the research questions? | Y |  |  |  |
|  | *Further appraisal may not be feasible or appropriate when the answer is ‘No’ or ‘Can’t tell’ to one or both screening questions.* | | |  | |
| 2. Quantitative randomized controlled  trials | 2.1. Is randomization appropriately performed? |  | N |  | Randomization was not appropriately described. |
|  | 2.2. Are the groups comparable at baseline? |  |  | 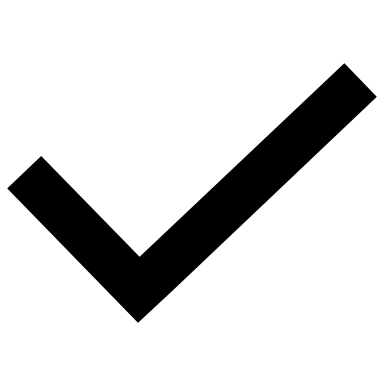 | baseline characteristics  and participants demographics not clear |
|  | 2.3. Are there complete outcome data? The drop out must be reposted. The good number is 15-25% |  |  | 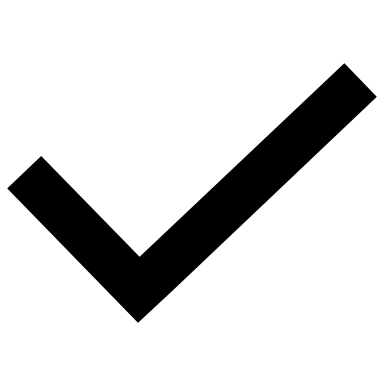 | withdrawal/dropouts rates not mentioned |
|  | 2.4. Are outcome assessors blinded to the intervention provided? |  |  | 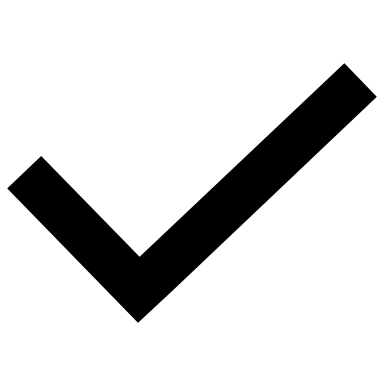 |  |
|  | 2.5 Did the participants adhere to the assigned intervention? | Y |  |  |  |

| [1] A. H. Ahmed, A. M. Roumani, K. Szucs, L. Zhang, and D. King, “The Effect of Interactive Web-Based Monitoring on Breastfeeding Exclusivity, Intensity, and Duration in Healthy, Term Infants After Hospital Discharge,” *JOGNN - J. Obstet. Gynecol. Neonatal Nurs.*, vol. 45, no. 2, pp. 143–154, 2016. | | | | | |
| --- | --- | --- | --- | --- | --- |
| **Category of study designs** | **Methodological quality criteria** |  | | **Responses** | |
|  |  | Yes | No | Can’t tell | Comments |
| Screening questions (for all types) | S1. Are there clear research questions? | Y |  |  |  |
|  | S2. Do the collected data allow to address the research questions? | Y |  |  |  |
|  | *Further appraisal may not be feasible or appropriate when the answer is ‘No’ or ‘Can’t tell’ to one or both screening questions.* | | |  | |
| 2. Quantitative randomized controlled  trials | 2.1. Is randomization appropriately performed? | Y |  |  | ﻿Participants were allocated ﻿by computer-generated random numbers using mode of delivery and parity as stratifying factors to control for these variables |
|  | 2.2. Are the groups comparable at baseline? | Y |  |  | *﻿*no significant difference between the control and intervention groups in the mothers’ demographic information |
|  | 2.3. Are there complete outcome data? | Y |  |  | The researchers added 30 additional mothers to the intervention group to allow for an attrition rate of 35% |
|  | 2.4. Are outcome assessors blinded to the intervention provided? |  |  | 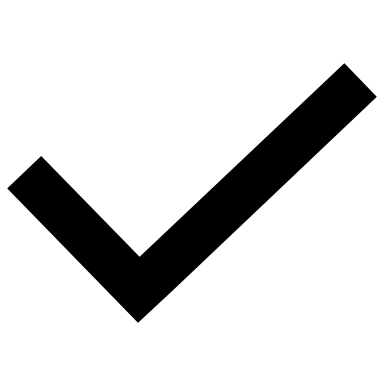 |  |
|  | 2.5 Did the participants adhere to the assigned intervention? | Y |  |  | 96% response rate for the first-month survey among the control group (n=57), with 91% and 80% response rates for the second and third months, respectively; the response rates for the first, second, and third months among the intervention group were 100%, 92%, and 88%, respectively (n=49). |

| [1] M. Z. Huang, S. C. Kuo, M. D. Avery, W. Chen, K. C. Lin, and M. L. Gau, “Evaluating effects of a prenatal web-based breastfeeding education programme in Taiwan,” *J. Clin. Nurs.*, vol. 16, no. 8, pp. 1571–1579, Aug. 2007. | | | | | |
| --- | --- | --- | --- | --- | --- |
| **Category of study designs** | **Methodological quality criteria** |  | | **Responses** | |
|  |  | Yes | No | Can’t tell | Comments |
| Screening questions (for all types) | S1. Are there clear research questions? | Y |  |  |  |
|  | S2. Do the collected data allow to address the research questions? | Y |  |  |  |
|  | *Further appraisal may not be feasible or appropriate when the answer is ‘No’ or ‘Can’t tell’ to one or both screening questions.* | | |  | |
| 3. Quantitative nonrandomized | 3.1. Are the participants representative of the target population? | Y |  |  | The target population was women at 29–36 weeks gestation using the Internet on regular basis. ﻿Statistical power analysis was used to calculate the required sample size. |
|  | 3.2. Are measurements appropriate regarding both the outcome and intervention (or exposure)? | Y |  |  | *﻿Pilot testing was conducted to determine the readability and clarity of the instruments.* |
|  | 3.3. Are there complete outcome data? | Y |  |  | Acceptable attrition  rate of 10% have been ﻿measured; ﻿  the dropout rate was 7.7% in each group |
|  | 3.4. Are the confounders accounted for in the design and analysis? | 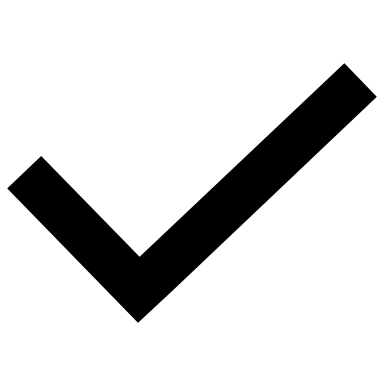 |  |  | no confounding expected  (double blinding procedure) |
|  | 3.5. During the study period, is the intervention administered (or exposure occurred) as intended? | Y |  |  | The researchers ﻿carried out the double blinding procedure. |

| [1] L. S. Hannula, M. E. Kaunonen, and P. J. Puukka, “A study to promote breast feeding in the Helsinki Metropolitan area in Finland,” *Midwifery*, vol. 30, no. 6, pp. 696–704, 2014. | | | | | |
| --- | --- | --- | --- | --- | --- |
| **Category of study designs** | **Methodological quality criteria** |  | | **Responses** | |
|  |  | Yes | No | Can’t tell | Comments |
| Screening questions (for all types) | S1. Are there clear research questions? | Y |  |  |  |
|  | S2. Do the collected data allow to address the research questions? | Y |  |  |  |
|  | *Further appraisal may not be feasible or appropriate when the answer is ‘No’ or ‘Can’t tell’ to one or both screening questions.* | | |  | |
| 3. Quantitative nonrandomized | 3.1. Are the participants representative of the target population? | Y |  |  | Study data was from a convenience  sample of mothers; clearly defined inclusion/﻿exclusion criteria |
|  | 3.2. Are measurements appropriate regarding both the outcome and intervention (or exposure)? | Y |  |  | ﻿﻿The﻿ instruments had been used previously and had been validated and pilot-tested |
|  | 3.3. Are there complete outcome data? |  | N |  | fairly low response rate to the questionnaires and the LATCH assessment |
|  | 3.4. Are the confounders accounted for in the design and analysis? | Y |  |  | (﻿It is possible that a greater number of multiparas with positive attitudes towards breast feeding may have been selected to the control group) |
|  | 3.5. During the study period, is the intervention administered (or exposure occurred) as intended? | Y |  |  |  |

| [1] A. H. Salonen, M. Kaunonen, P. Åstedt-Kurki, A. L. Järvenpää, and M. T. Tarkka, “Development of an internet-based intervention for parents of infants,” *J. Adv. Nurs.*, vol. 64, no. 1, pp. 60–72, 2008. | | | | | |
| --- | --- | --- | --- | --- | --- |
| **Category of study designs** | **Methodological quality criteria** |  | | Responses | |
|  |  | Yes | No | Can’t tell | Comments |
| Screening questions (for all types) | S1. Are there clear research questions? | Y |  |  |  |
|  | S2. Do the collected data allow to address the research questions? | Y |  |  |  |
|  | *Further appraisal may not be feasible or appropriate when the answer is ‘No’ or ‘Can’t tell’ to one or both screening questions.* | | |  | |
| 3. Quantitative nonrandomized | 3.1. Are the participants representative of the target population? | Y |  |  | 1300 sample with clear inclusion/exclusion criteria |
|  | 3.2. Are measurements appropriate regarding both the outcome and intervention (or exposure)? | Y |  |  | ﻿internal consistency reliability of measures is available |
|  | 3.3. Are there complete outcome data? | Y |  |  | Low refusal rate |
|  | 3.4. Are the confounders accounted for in the design and analysis? | Y |  |  | ﻿confounding variables are controlled |
|  | 3.5. During the study period, is the intervention administered (or exposure occurred) as intended? | Y |  |  |  |

| [2] A. H. Salonen, K. F. Pridham, R. L. Brown, and M. Kaunonen, “Impact of an internet-based intervention on Finnish mothers’ perceptions of parenting satisfaction, infant centrality and depressive symptoms during the postpartum year,” *Midwifery*, vol. 30, no. 1, pp. 112–122, 2014. | | | | | |
| --- | --- | --- | --- | --- | --- |
| **Category of study designs** | **Methodological quality criteria** |  | | **Responses** | |
|  |  | Yes | No | Can’t tell | Comments |
| Screening questions (for all types) | S1. Are there clear research questions? | Y |  |  |  |
|  | S2. Do the collected data allow to address the research questions? | Y |  |  |  |
|  | *Further appraisal may not be feasible or appropriate when the answer is ‘No’ or ‘Can’t tell’ to one or both screening questions.* | | |  | |
| 3. Quantitative nonrandomized | 3.1. Are the participants representative of the target population? | Y |  |  | ﻿The sample (N=1300), ﻿power analysis was performed to determine the sample size needed |
|  | 3.2. Are measurements appropriate regarding both the outcome and intervention (or exposure)? | Y |  |  | ﻿general linear mixed modelling longitudinal analysis  was used to assess the outcomes  ﻿﻿Intent-to-treat (ITT) analysis was used to compare the mothers in the target hospitals |
|  | 3.3. Are there complete outcome data? | Y |  |  | A pattern mixture analysis was used to examine missingness and dropouts. |
|  | 3.4. Are the confounders accounted for in the design and analysis? |  |  | 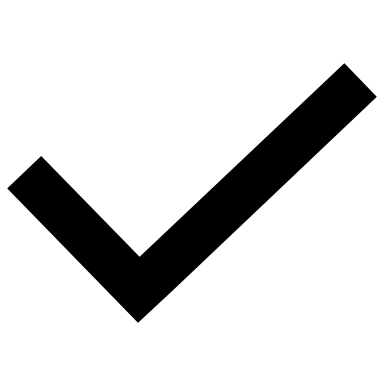 | Comparison of the characteristics of participants indicated that intervention mothers were somewhat older, more educated and first-time mothers more often compared to the controls. |
|  | 3.5. During the study period, is the intervention administered (or exposure occurred) as intended? | Y |  |  | The intervention was developed and implemented in the intervention hospital. |

| [1] N. Geoghegan-Morphet, D. Yuen, E. Rai, M. Angelini, M. Christmas, and O. da Silva, “Development and Implementation of a Novel Online Breastfeeding Support Resource: The Maternal Virtual Infant Nutrition Support Clinic,” *Breastfeed. Med.*, vol. 9, no. 10, pp. 520–523, 2014. | | | | | |
| --- | --- | --- | --- | --- | --- |
| **Category of study designs** | **Methodological quality criteria** |  | | **Responses** | |
|  |  | Yes | No | Can’t tell | Comments |
| Screening questions (for all types) | S1. Are there clear research questions? | Y |  |  | **Hypothesis was proposed** |
|  | S2. Do the collected data allow to address the research questions? | Y |  |  |  |
|  | *Further appraisal may not be feasible or appropriate when the answer is ‘No’ or ‘Can’t tell’ to one or both screening questions.* | | |  | |
| 1. Qualitative | 1.1. Is the qualitative approach appropriate to answer the research question? | Y |  |  | (**Qualitative hypothesis** testing)  collecting survey and feedback data from participants to test the **hypothesis** |
|  | 1.2. Are the qualitative data collection methods adequate to address the research question? | Y |  |  | ﻿Collected data on breastfeeding outcomes from survey and feedback |
|  | 1.3. Are the findings adequately derived from the data? |  | N |  | Data collected by ﻿  Web site analytics-no indication of any data analysis method |
|  | 1.4. Is the interpretation of results sufficiently substantiated by data? | Y |  |  |  |
|  | 1.5. Is there coherence between qualitative data sources, collection, analysis and interpretation? |  | N |  | Data analysis process and interpretation of the data are not clear |

| [1] J. S. Grassley, K. C. Connor, and L. Bond, “Game-based online antenatal breastfeeding education: A pilot,” *Appl. Nurs. Res.*, vol. 33, pp. 93–95, 2017. | | | | | |
| --- | --- | --- | --- | --- | --- |
| **Category of study designs** | **Methodological quality criteria** |  | | **Responses** | |
|  |  | Yes | No | Can’t tell | Comments |
| Screening questions (for all types) | S1. Are there clear research questions? | Y |  |  |  |
|  | S2. Do the collected data allow to address the research questions? | Y |  |  |  |
|  | *Further appraisal may not be feasible or appropriate when the answer is ‘No’ or ‘Can’t tell’ to one or both screening questions.* | | |  | |
| 4. Quantitative descriptive | 4.1. Is the sampling strategy relevant to address the research question? |  |  | 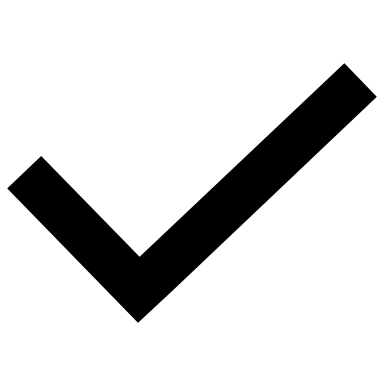 | ﻿convenience sample of 41women |
|  | 4.2. Is the sample representative of the target population? |  | N |  | No clear ﻿description of the target population |
|  | 4.3. Are the measurements appropriate? | Y |  |  | *﻿*Variables are appropriately defined and measured |
|  | 4.4. Is the risk of nonresponse bias low? |  |  | 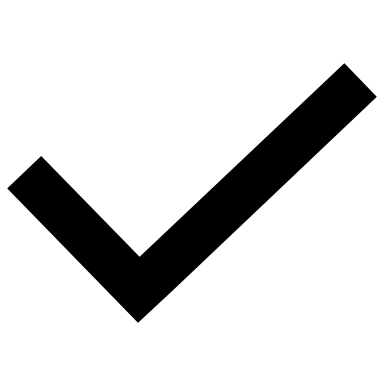 | Not mentioned in the study |
|  | 4.5. Is the statistical analysis appropriate to answer the research question? | Y |  |  | ﻿Descriptive statistics and ﻿one-way ANOVA |

| [1] A. H. Ahmed and M. Ouzzani, “Interactive Web-Based Breastfeeding Monitoring,” *J. Hum. Lact.*, vol. 28, no. 4, pp. 468–475, 2012. | | | | | |
| --- | --- | --- | --- | --- | --- |
| **Category of study designs** | **Methodological quality criteria** |  | | **Responses** | |
|  |  | Yes | No | Can’t tell | Comments |
| Screening questions (for all types) | S1. Are there clear research questions? | Y |  |  |  |
|  | S2. Do the collected data allow to address the research questions? | Y |  |  |  |
|  | *Further appraisal may not be feasible or appropriate when the answer is ‘No’ or ‘Can’t tell’ to one or both screening questions.* | | |  | |
| 5. Mixed methods | 5.1. Is there an adequate rationale for using a mixed methods design to address the research question? |  | N |  | No clearly explained reasons for conducting a mixed methods study |
|  | 5.2. Are the different components of the study effectively integrated to answer the research question? | Y |  |  | *descriptive statistics were used to examine the sample and analyse the feasibility and usability data(quantitative data).*  *﻿Mothers’ responses to the Perception Survey were analysed with ﻿*  *qualitative content analysis* |
|  | 5.3. Are the outputs of the integration of qualitative and quantitative components adequately interpreted? | Y |  |  |  |
|  | 5.4. Are divergences and inconsistencies between quantitative and qualitative results adequately addressed? |  |  | 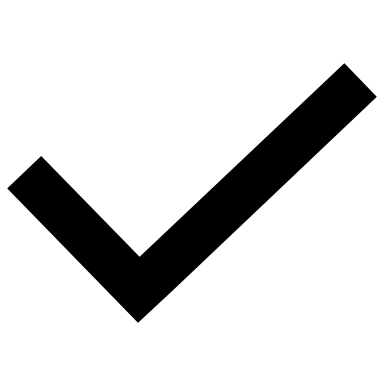 |  |
|  | 5.5. Do the different components of the study adhere to the quality criteria of each tradition of the methods involved? | Y |  |  |  |

| [1] R. Giglia, K. Cox, Y. Zhao, and C. W. Binns, “Exclusive breastfeeding increased by an internet intervention,” *Breastfeed. Med.*, vol. 10, no. 1, pp. 20–25, Feb. 2015. | | | | | |
| --- | --- | --- | --- | --- | --- |
| **Category of study designs** | **Methodological quality criteria** |  | | **Responses** | |
|  |  | Yes | No | Can’t tell | Comments |
| Screening questions (for all types) | S1. Are there clear research questions? | Y |  |  |  |
|  | S2. Do the collected data allow to address the research questions? | YY |  |  |  |
|  | *Further appraisal may not be feasible or appropriate when the answer is ‘No’ or ‘Can’t tell’ to one or both screening questions.* | | |  | |
| 3. Quantitative nonrandomized | 3.1. Are the participants representative of the target population? | Y |  |  | Mothers were recruited for a period of 21 months  to obtain the  required sample size. |
|  | 3.2. Are measurements appropriate regarding both the outcome and intervention (or exposure)? | Y |  |  | ﻿Univariate summary statistics |
|  | 3.3. Are there complete outcome data? | Y |  |  | ﻿(85%) women were enrolled in the Internet study |
|  | 3.4. Are the confounders accounted for in the design and analysis? | Y |  |  | Participants were randomly assigned, demographic characteristics for each group did  not differ significantly |
|  | 3.5. During the study period, is the intervention administered (or exposure occurred) as intended? | Y |  |  |  |

| [1] C.-J. Wang, P. Chaovalit, and S. Pongnumkul, “A Breastfeed-Promoting Mobile App Intervention: Usability and Usefulness Study,” *JMIR mHealth uHealth*, vol. 6, no. 1, p. e27, 2018. | | | | | |
| --- | --- | --- | --- | --- | --- |
| **Category of study designs** | **Methodological quality criteria** |  | | **Responses** | |
|  |  | Yes | No | Can’t tell | Comments |
| Screening questions (for all types) | S1. Are there clear research questions? | Y |  |  |  |
|  | S2. Do the collected data allow to address the research questions? | Y |  |  |  |
|  | *Further appraisal may not be feasible or appropriate when the answer is ‘No’ or ‘Can’t tell’ to one or both screening questions.* | | | *.* | |
| 5. Mixed methods | 5.1. Is there an adequate rationale for using a mixed methods design to address the research question? |  | N |  |  |
|  | 5.2. Are the different components of the study effectively integrated to answer the research question? | Y |  |  |  |
|  | 5.3. Are the outputs of the integration of qualitative and quantitative components adequately interpreted? | Y |  |  |  |
|  | 5.4. Are divergences and inconsistencies between quantitative and qualitative results adequately addressed? |  | N |  |  |
|  | 5.5. Do the different components of the study adhere to the quality criteria of each tradition of the methods involved? |  |  | 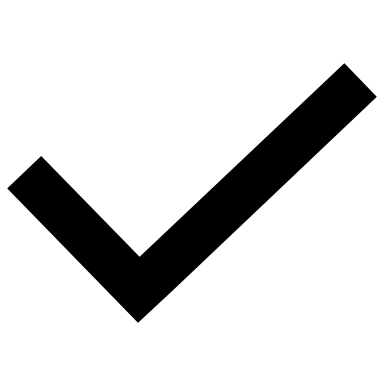 |  |

| [1] D. R. Dela Cruz and D. M. M. Mendoza, “Milktrack: Design and development of mobile application and logistics system in empowering breastfeeding practice in the Philippines,” in *IEEE Region 10 Annual International Conference, Proceedings/TENCON*, 2017, vol. 2017-Decem, pp. 2242–2246. | | | | | |
| --- | --- | --- | --- | --- | --- |
| **Category of study designs** | **Methodological quality criteria** |  | | **Responses** | |
|  |  | Yes | No | Can’t tell | Comments |
| Screening questions (for all types) | S1. Are there clear research questions? |  | N |  |  |
|  | S2. Do the collected data allow to address the research questions? |  |  | 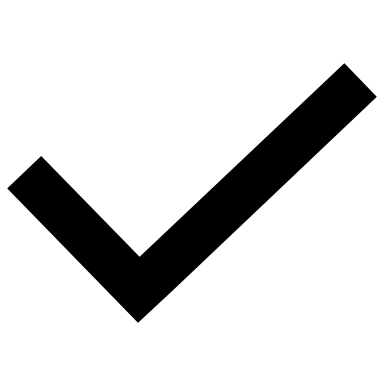 |  |
|  | *Further appraisal may not be feasible or appropriate when the answer is ‘No’ or ‘Can’t tell’ to one or both screening questions.* | | |  | |
| 1. Qualitative | 1.1. Is the qualitative approach appropriate to answer the research question? |  |  | 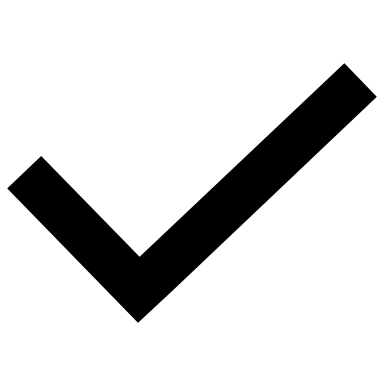 |  |
|  | 1.2. Are the qualitative data collection methods adequate to address the research question? |  | N |  |  |
|  | 1.3. Are the findings adequately derived from the data? | Y |  |  | From questionnaire to (32) mothers |
|  | 1.4. Is the interpretation of results sufficiently substantiated by data? |  | N |  |  |
|  | 1.5. Is there coherence between qualitative data sources, collection, analysis and interpretation? |  | N |  |  |

| [1] N. Wheaton, J. Lenehan, and L. H. Amir, “Evaluation of a Breastfeeding App in Rural Australia: Prospective Cohort Study,” *J. Hum. Lact.*, vol. 34, no. 4, pp. 711–720, Nov. 2018. | | | | | |
| --- | --- | --- | --- | --- | --- |
| **Category of study designs** | **Methodological quality criteria** |  | | **Responses** | |
|  |  | Yes | No | Can’t tell | Comments |
| Screening questions (for all types) | S1. Are there clear research questions? | Y |  |  |  |
|  | S2. Do the collected data allow to address the research questions? | Y |  |  |  |
|  | *Further appraisal may not be feasible or appropriate when the answer is ‘No’ or ‘Can’t tell’ to one or both screening questions.* | | |  | |
| 3. Quantitative nonrandomized | 3.1. Are the participants representative of the target population? | Y |  |  | ﻿The study sample was a convenience sample |
|  | 3.2. Are measurements appropriate regarding both the outcome and intervention (or exposure)? | Y |  |  | ﻿descriptive statistics |
|  | 3.3. Are there complete outcome data? |  |  | 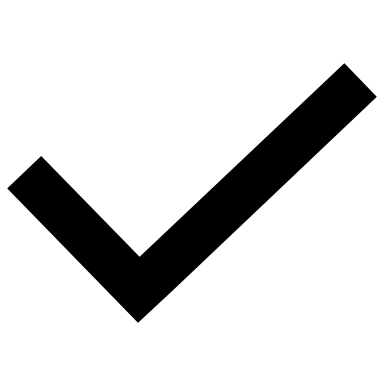 | ﻿Of the initial participants (N = 46), 63% of participants (n = 29) completed both surveys at 3 and 6 months. |
|  | 3.4. Are the confounders accounted for in the design and analysis? | Y |  |  | *﻿*The demographic characteristics of the ﻿participants at baseline were similar to those of participants who completed all data collection points |
|  | 3.5. During the study period, is the intervention administered (or exposure occurred) as intended? | Y |  |  |  |

| [1] G. Alberdi *et al.*, “A feasibility study of a multidimensional breastfeeding-support intervention in Ireland,” *Midwifery*, vol. 58, no. July 2017, pp. 86–92, 2018. | | | | | |
| --- | --- | --- | --- | --- | --- |
| **Category of study designs** | **Methodological quality criteria** |  | | **Responses** | |
|  |  | Yes | No | Can’t tell | Comments |
| Screening questions (for all types) | S1. Are there clear research questions? | Y |  |  |  |
|  | S2. Do the collected data allow to address the research questions? | Y |  |  |  |
|  | *Further appraisal may not be feasible or appropriate when the answer is ‘No’ or ‘Can’t tell’ to one or both screening questions.* | | |  | |
| 4. Quantitative descriptive | 4.1. Is the sampling strategy relevant to address the research question? |  |  | 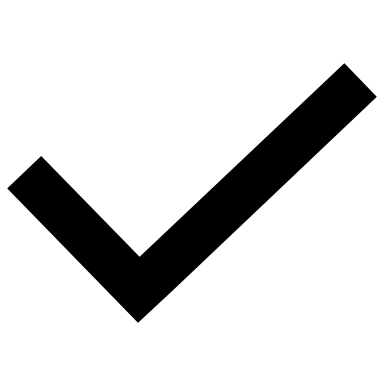 | No clear justification of the sample frame |
|  | 4.2. Is the sample representative of the target population? | Y |  |  | Provided reasons for those who declined to participate |
|  | 4.3. Are the measurements appropriate? | Y |  |  | *﻿*Categorical data are summarised in frequency tables using counts  and percentages. |
|  | 4.4. Is the risk of nonresponse bias low? |  | N |  | *﻿*100 women participated in the intervention. 76 women had a one-to-one postnatal consultation with a lactation consultant in Dublin and 23 in Wexford. 50 and 45 women in Dublin, and 15 and 15 in Wexford responded to the 6-week and 3-month questionnaires, respectively. |
|  | 4.5. Is the statistical analysis appropriate to answer the research question? | Y |  |  |  |

| [1] R. Newby, W. Brodribb, R. S. Ware, and P. S. W. Davies, “Internet use by first-time mothers for infant feeding support,” *J. Hum. Lact.*, vol. 31, no. 3, pp. 416–424, 2015. | | | | | |
| --- | --- | --- | --- | --- | --- |
| **Category of study designs** | **Methodological quality criteria** |  | | **Responses** | |
|  |  | Yes | No | Can’t tell | Comments |
| Screening questions (for all types) | S1. Are there clear research questions? | Y |  |  |  |
|  | S2. Do the collected data allow to address the research questions? | Y |  |  |  |
|  | *Further appraisal may not be feasible or appropriate when the answer is ‘No’ or ‘Can’t tell’ to one or both screening questions.* | | |  | |
| 3. Quantitative nonrandomized | 3.1. Are the participants representative of the target population? | Y |  |  | ﻿recruited into the Study by convenience sampling |
|  | 3.2. Are measurements appropriate regarding both the outcome and intervention (or exposure)? | Y |  |  | *﻿*univariable  regression was conducted, and then multivariable analyses adjusting for maternal age and SES were undertaken. |
|  | 3.3. Are there complete outcome data? |  |  | 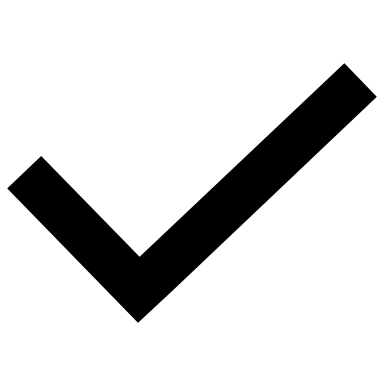 | No acceptable dropouts rates have been suggested |
|  | 3.4. Are the confounders accounted for in the design and analysis? |  | N |  | No appropriate methods to control for confounders are used |
|  | 3.5. During the study period, is the intervention administered (or exposure occurred) as intended? | Y |  |  |  |
