# Supplementary material for: Effectiveness of Internet-Based Electronic Technology Interventions on Breastfeeding Outcomes: Systematic Review
Source: J Med Internet Res. 2020 May 29;22(5):e17361. doi: 10.2196/17361 (PMC7293063; doi:10.2196/17361)
Supplement: Multimedia Appendix 5 [file jmir_v22i5e17361_app5.docx]

**Multimedia Appendix: Excluded studies**

To assess eligibility, full text of 52 articles was reviewed by 2 reviewers. The reason for exclusion was described as listed below, though several articles had multiple reasons for exclusion.

| **Authors (publication year)** | **Reason for exclusion** |
| --- | --- |
| Wrong intervention | |
| Seguranyes, G. *et al.* (2014) | Virtual consultation  (consult midwives by videoconference or telephone) |
| Mohamadirizi, S., Bahadoran, P. and Fahami F, F. (2014) | education via E-learning with no internet access |
| Laborde, L. *et al.* (2007) | CD-ROM-based program |
| Labarère, J. *et al.* (2011) | CD-ROM-based program |
| Fahami, F., Mohamadirizi, S., Bahadoran, P. (2014) | electronic education with no internet access |
| Alnasser, Y. et al. (2018) | educational video with no internet access |
| Reeder, J. A. *et al.* (2014) | telephone peer counselling program |
| Rojjanasrirat, W., Nelson, E. L. and Wambach, K. A. (2012) | videoconferencing sessions to deliver lactation support |
| Alam, M. *et al.* (2017) | mobile phone-based messaging services intervention with no internet access |
| Harris-Luna, M. L. and Badr, L. K. (2018) | telephone support for breastfeeding |
| Prieto, J. T., Zuleta, C. and Rodríguez, J. T. (2017) | health-promoting text messages |
| Gallegos, D., Cromack, C. and Thorpe, K. J. (2018) | 24-hour telephone support resource |
| Meedya, S. *et al.* (2014) | telephone support for breastfeeding |
| Martín Jiménez, M. del P. *et al.* (2018) | telephone support for breastfeeding |
| Gu, Y. *et al.* (2016) | intervention programme:  individual instruction, group education and telephone counselling. |
| Olson, B. H. *et al.* (2010) | ﻿telephone contacts |
| Khresheh, R. *et al.* (2011) | pamphlet containing information about breastfeeding and follow-up phone calls |
| McIlvennan, C. K., Eapen, Z. J. and Allen, L. A. (2015) | telephone delivered educational session and phone/text access to a nurse |
| Unger, J. A. *et al.* (2018) | SMS (Mobile SMS delivery platform) |
| Dennis, C. L. *et al.* (2002) | telephone-based peer support intervention |
| Martinez-Brockman, J. L. *et al.* (2018) | text messaging intervention |
| Harari, N. *et al.* (2018) | text message intervention |
| Edwards, R. A. *et al.* (2013) | ﻿computer-based animated, interactive agent with no internet access |
| Awano, M. and Shimada, K. (2010) | pamphlet and a DVD intervention |
| Gallegos, D. *et al.* (2014) | text messaging service |
| M., B. *et al.* (2010) | telephone calls by nurses |
| Jiang, H. *et al.* (2014) | SMS messages |
| Tahir, N. M. and Al-Sadat, N. (2013) | lactation counselling via telephone |
| Flax, V. L. *et al.* (2017) | breastfeeding voice and text messages with group cell phones members |
| Flax, V. L. *et al.* (2014) | cell phone text and voice messages |
| Kellams, A. L. et al. (2016) | view an educational video with no internet access |
| No study data reported | |
| Clark, A. *et al.* (2009) | Changes in attitudes and behaviours were reported with no breastfeeding data |
| Demirci, J. R. *et al.* (2017) | Only tested the feasibility of a breastfeeding tracking app and do not report any breastfeeding data |

**Reference list for excluded studies**

Meedya, S. 2015, *Increasing breastfeeding rates up to six months among primparous women : a quasi-experimental study of the effectiveness of the Milky Way Program Increasing Breastfeeding Rates Up to Six Months among Primiparous Women : A Quasi-Experimental Study of the E*, no. May.

Laborde, L., Gelbert-Baudino, N., Fulcheri, J., Schelstraete, C., Francois, P. & Labarere, J. 2007, ‘Breastfeeding outcomes for mothers with and without home access to e-technologies’, *Acta Paediatrica, International Journal of Paediatrics*, vol. 96, no. 7, pp. 1071–5, viewed 21 April 2019, <http://doi.wiley.com/10.1111/j.1651-2227.2007.00369.x>.

Alnasser, Y., Almasoud, N., Aljohni, D., Almisned, R., Alsuwaine, B., Alohali, R., Almutairi, O. & Alhezayen, R. 2018, ‘Impact of attitude and knowledge on intention to breastfeed: Can mHealth based education influence decision to breastfeed exclusively?’, *Annals of Medicine and Surgery*, vol. 35, pp. 6–12, viewed 16 November 2018, <https://doi.org/10.1016/j.amsu.2018.09.007>.

Unger, J.A., Ronen, K., Perrier, T., DeRenzi, B., Slyker, J., Drake, A.L., Mogaka, D., Kinuthia, J. & John-Stewart, G. 2018, ‘Short message service communication improves exclusive breastfeeding and early postpartum contraception in a low- to middle-income country setting: a randomised trial’, *BJOG: An International Journal of Obstetrics and Gynaecology*, vol. 125, no. 12, pp. 1620–9.

Alam, M., D’Este, C., Banwell, C. & Lokuge, K. 2017, ‘The impact of mobile phone based messages on maternal and child healthcare behaviour: A retrospective cross-sectional survey in Bangladesh’, *BMC Health Services Research*, vol. 17, no. 1, viewed 19 November 2018, <https://bmchealthservres.biomedcentral.com/track/pdf/10.1186/s12913-017-2361-6?site=bmchealthservres.biomedcentral.com>.

Gallegos, D., Russell-Bennett, R., Previte, J. & Parkinson, J. 2014, ‘Can a text message a week improve breastfeeding?’, *BMC Pregnancy and Childbirth*, vol. 14, no. 1, p. 374.

McIlvennan, C.K., Eapen, Z.J. & Allen, L.A. 2015, ‘Hospital readmissions reduction program’, *Circulation*, vol. 131, no. 20, pp. 1796–803.

Da Rocha, S.T., Charalambous, M., Lin, S.P., Gutteridge, I., Ito, Y., Gray, D., Dean, W. & Ferguson-Smith, A.C. 2009, ‘Gene dosage effects of the imprinted delta-like homologue 1 (Dlk1/Pref1) in development: Implications for the evolution of imprinting’, *PLoS Genetics*, vol. 5, no. 2, pp. 21–8, viewed 4 November 2018, <https://www.ncbi.nlm.nih.gov/pmc/articles/PMC99222/pdf/20020108s00013p21.pdf>.

Fahami, F., Mohamadirizi, S., Bahadoran, P. 2014, ‘Effect of Electronic Education o the awareness of women post-partum breastfeeding’, *Internataional Journal of Pediatrics*, vol. 2, no. 8, pp. 57–63.

Awano, M. & Shimada, K. 2010, ‘Development and evaluation of a self care program on breastfeeding in Japan: A quasi-experimental study’, *International Breastfeeding Journal*, vol. 5, no. 1, p. 9.

Khresheh, R., Suhaimat, A., Jalamdeh, F. & Barclay, L. 2011, ‘The effect of a postnatal education and support program on breastfeeding among primiparous women: A randomized controlled trial’, *International Journal of Nursing Studies*, vol. 48, no. 9, pp. 1058–65.

Tahir, N.M. & Al-Sadat, N. 2013, ‘Does telephone lactation counselling improve breastfeeding practices?: A randomised controlled trial’, *International Journal of Nursing Studies*, vol. 50, no. 1, pp. 16–25.

Jiang, H., Li, M., Wen, L.M., Hu, Q., Yang, D., He, G., Baur, L.A., Dibley, M.J. & Qian, X. 2014, ‘Effect of short message service on infant feeding practice findings from a community-based study in shanghai, china’, *JAMA Pediatrics*, vol. 168, no. 5, pp. 471–8.

Sutherland, M., Fantasia, H.C. & Fontenot, H.B. 2015, ‘Telephone support for breastfeeding by primary care: A randomised multicentre trial’, *JOGNN - Journal of Obstetric, Gynecologic, and Neonatal Nursing*, vol. 44, p. S56, viewed 28 November 2018, <https://doi.org/10.1016/j.anpedi.2018.02.007>.

Harris-Luna, M.L. & Badr, L.K. 2018, ‘Pragmatic Trial to Evaluate the Effect of a Promotora Telephone Intervention on the Duration of Breastfeeding’, *JOGNN - Journal of Obstetric, Gynecologic, and Neonatal Nursing*, vol. 47, no. 6, pp. 738–48, viewed 19 November 2018, <https://doi.org/10.1016/j.jogn.2018.09.001>.

Gallegos, D., Cromack, C. & Thorpe, K.J. 2018, ‘Can a phone call make a difference? Breastfeeding self-efficacy and nurse responses to mother’s calls for help’, *Journal of Child Health Care*, vol. 22, no. 3, pp. 433–46, viewed 19 November 2018, <https://journals-sagepub-com.ezproxy.uow.edu.au/doi/pdf/10.1177/1367493518757066>.

Mohamadirizi, S., Bahadoran, P. & Fahami F, F. 2014, ‘Effect of E-learning on primigravida women′s satisfaction and awareness concerning prenatal care’, *Journal of Education and Health Promotion*, vol. 3, no. 1, p. 13.

Kellams, A.L., Gurka, K.K., Hornsby, P.P., Drake, E., Riffon, M., Gellerson, D., Gulati, G. & Coleman, V. 2016, ‘The Impact of a Prenatal Education Video on Rates of Breastfeeding Initiation and Exclusivity during the Newborn Hospital Stay in a Low-income Population’, *Journal of Human Lactation*.

Rojjanasrirat, W., Nelson, E.-L. & Wambach, K.A. 2012, ‘A pilot study of home-based videoconferencing for breastfeeding support’, *Journal of Human Lactation*, vol. 28, no. 4, pp. 464–7.

Clark, A., Anderson, J., Adams, E., Baker, S. & Barrett, K. 2009, ‘Assessing an Infant Feeding Web Site as a Nutrition Education Tool for Child Care Providers’, *Journal of Nutrition Education and Behavior*, vol. 41, no. 1, pp. 41–6.

Martinez-Brockman, J.L., Harari, N., Segura-Pérez, S., Goeschel, L., Bozzi, V. & Pérez-Escamilla, R. 2018, ‘Impact of the Lactation Advice Through Texting Can Help (LATCH) Trial on Time to First Contact and Exclusive Breastfeeding among WIC Participants’, *Journal of Nutrition Education and Behavior*, vol. 50, no. 1, pp. 33-42.e1, viewed 16 November 2018, <https://doi.org/10.1016/j.jneb.2017.09.001>.

Prieto, J.T., Zuleta, C. & Rodríguez, J.T. 2017, ‘Modeling and testing maternal and newborn care mHealth interventions: A pilot impact evaluation and follow-up qualitative study in Guatemala’, *Journal of the American Medical Informatics Association*.

Olson, B.H., Haider, S.J., Vangjel, L., Bolton, T.A. & Gold, J.G. 2010, ‘A quasi-experimental evaluation of a breastfeeding support program for low income women in Michigan’, *Maternal and Child Health Journal*, vol. 14, no. 1, pp. 86–93.

Flax, V.L., Ibrahim, A.U., Negerie, M., Yakubu, D., Leatherman, S. & Bentley, M.E. 2017, ‘Group cell phones are feasible and acceptable for promoting optimal breastfeeding practices in a women’s microcredit program in Nigeria’, *Maternal and Child Nutrition*, vol. 13, no. 1.

Labarère, J., Gelbert-Baudino, N., Laborde, L., Arragain, D., Schelstraete, C. & François, P. 2011, ‘CD-ROM-based program for breastfeeding mothers’, *Maternal and Child Nutrition*, vol. 7, no. 3, pp. 263–72.

Harari, N., Rosenthal, M.S., Bozzi, V., Goeschel, L., Jayewickreme, T., Onyebeke, C., Griswold, M. & Perez-Escamilla, R. 2018, ‘Feasibility and acceptability of a text message intervention used as an adjunct tool by WIC breastfeeding peer counsellors: The LATCH pilot’, *Maternal and Child Nutrition*, vol. 14, no. 1.

Demirci, J.R. & Bogen, D.L. 2017, ‘Feasibility and acceptability of a mobile app in an ecological momentary assessment of early breastfeeding’, *Maternal and Child Nutrition*, vol. 13, no. 3, viewed 31 October 2018, <https://www.ncbi.nlm.nih.gov/pmc/articles/PMC5237417/pdf/nihms819140.pdf>.

Seguranyes, G., Costa, D., Fuentelsaz-Gallego, C., Beneit, J.V., Carabantes, D., Gómez-Moreno, C., Palacio-Tauste, A., Pauli, A., Abella, M., Seguranyes, G., Gómez-Moreno, C., Terré, C., Viñas, H., Costa, D., Martínez-Bueno, C., Falguera, G., Pauli, A., León, C., Gol-Gómez, R., Palacio-Tauste, A., Gutierrez-Culsan, I., Duran-Moyano, N., Bru-Serra, E., Romano, E., Pinto, T., Abella, M., Miralpeix, G., Martínez-Juan, J., Zaragoza, A., Adell, R.M., Peñas, J., Guix, D., Relat Llavina, J., Corchs Cutura, S., Santaella, M., Aliaga, F., Prats, E., Lladó, M., Barnes, E., Hernández, M., Redondo, L., Valls-Antón, O. & Miguel, S. 2014, ‘Efficacy of a videoconferencing intervention compared with standard postnatal care at primary care health centres in Catalonia’, *Midwifery*, vol. 30, no. 6, pp. 764–71.

Gu, Y., Zhu, Y., Zhang, Z. & Wan, H. 2016, ‘Effectiveness of a theory-based breastfeeding promotion intervention on exclusive breastfeeding in China: A randomised controlled trial’, *Midwifery*.

Reeder, J.A., Joyce, T., Sibley, K., Arnold, D. & Altindag, O. 2014, ‘Telephone peer counseling of breastfeeding among WIC participants: A randomized controlled trial’, *Pediatrics*, vol. 134, no. 3, pp. e700–9.

Flax, V.L., Negerie, M., Ibrahim, A.U., Leatherman, S., Daza, E.J. & Bentley, M.E. 2014, ‘Integrating Group Counseling, Cell Phone Messaging, and Participant-Generated Songs and Dramas into a Microcredit Program Increases Nigerian Women’s Adherence to International Breastfeeding Recommendations’, *The Journal of Nutrition*.
